# Systematic assessment in an animal model of the angiogenic potential of different human cell sources for therapeutic revascularization

**DOI:** 10.1186/scrt114

**Published:** 2012-07-03

**Authors:** G Robin Barclay, Olga Tura, Kay Samuel, Patrick WF Hadoke, Nicholas L Mills, David E Newby, Marc L Turner

**Affiliations:** 1 SNBTS Cell Therapy Research Group, Scottish Centre for Regenerative Medicine, University of Edinburgh, 49 Little France Crescent, Edinburgh EH16 4SB, UK; 2 BHF/University Centre for Cardiovascular Science, University of Edinburgh, 49 Little France Crescent, Edinburgh EH16 4SB, UK

## Abstract

**Introduction:**

Endothelial progenitor cells (EPC) capable of initiating or augmenting vascular growth were recently identified within the small population of CD34-expressing cells that circulate in human peripheral blood and which are considered hematopoietic progenitor cells (HPC). Soon thereafter human HPC began to be used in clinical trials as putative sources of EPC for therapeutic vascular regeneration, especially in myocardial and critical limb ischemias. However, unlike HPC where hematopoietic efficacy is related quantitatively to CD34^+ ^cell numbers implanted, there has been no consensus on how to measure EPC or how to assess cellular graft potency for vascular regeneration. We employed an animal model of spontaneous neovascularization to simultaneously determine whether human cells incorporate into new vessels and to quantify the effect of different putative angiogenic cells on vascularization in terms of number of vessels generated. We systematically compared competence for therapeutic angiogenesis in different sources of human cells with putative angiogenic potential, to begin to provide some rationale for optimising cell procurement for this therapy.

**Methods:**

Human cells employed were mononuclear cells from normal peripheral blood and HPC-rich cell sources (umbilical cord blood, mobilized peripheral blood, bone marrow), CD34^+ ^enriched or depleted subsets of these, and outgrowth cell populations from these. An established sponge implant angiogenesis model was adapted to determine the effects of different human cells on vascularization of implants in immunodeficient mice. Angiogenesis was quantified by vessel density and species of origin by immunohistochemistry.

**Results:**

CD34^+ ^cells from mobilized peripheral blood or umbilical cord blood HPC were the only cells to promote new vessel growth, but did not incorporate into vessels. Only endothelial outgrowth cells (EOC) incorporated into vessels, but these did not promote vessel growth.

**Conclusions:**

These studies indicate that, since EPC are very rare, any benefit seen in clinical trials of HPC in therapeutic vascular regeneration is predominantly mediated by indirect proangiogenic effects rather than through direct incorporation of any rare EPC contained within these sources. It should be possible to produce autologous EOC for therapeutic use, and evaluate the effect of EPC distinct from, or in synergy with, the proangiogenic effects of HPC therapies.

## Introduction

Circulating endothelial progenitor cells (EPC) were first recognized in 1997 [1,2], introducing the concept that circulating EPC might supplement local angiogenesis which had heretofore been viewed as arising solely by outgrowth from pre-existing vasculature. Thus EPC had potential for development of cell-based therapeutic angiogenesis. EPC in adults were proposed to share a common stem cell with hematopoietic progenitor cells (HPC)[3], and like HPC express CD34 and mobilize from bone marrow [1,2]. It was proposed that, in the absence of a precise phenotype definition, EPC would coincide with HPC. Consequently, development of therapy progressed rapidly through preclinical studies to early clinical studies by employing HPC sources as therapeutic cells on the presumption that these contained EPC. It was shown that such procedures were safe and showed modest benefit in the treatment of myocardial and peripheral ischemia [4-6].

It was widely supposed that any therapeutic benefit was mainly achieved by delivery of EPC that home to sites of active angiogenesis where they proliferate and incorporate into new vasculature. If this is correct, efficacy should be related to the quantity of EPC delivered. However, it was recognised early that therapeutic angiogenesis is complex [5], and continuing studies of therapeutic angiogenesis by HPC in cardiac [7,8] and peripheral [9,10] ischemias have not shown consistent clinical efficacy. This lack of obvious clinical benefit has led to calls for a better understanding of the identities and roles of cells participating in angiogenesis where there is recognition of the distinct effects of direct participation (incorporation) and indirect promotion (paracrine effect), so that the cell-based therapies can be designed to be more beneficial[11,12]. This might be achieved by sourcing, enrichment and manipulation of appropriate effector cells when such cells and their roles can be defined. Reported clinical studies have all employed autologous bone marrow or mobilized peripheral blood HPC as the therapeutic source, either as unfractionated mononuclear cells (MNC) or as enriched HPC by selection of CD34^+ ^or CD133^+ ^MNC. However since the identity of EPC has been ambiguous, there can be no confidence that the most appropriate therapeutic cells have been employed. For example, the issue as to whether or not EPC express CD133 has been controversial, but is now resolving to indicate that EPC do not express CD133 [13-15], so it seems that some trials that have employed CD133-enriched HPC may not have delivered EPC in the implanted cells. Although a variety of sources and cell fractions have been employed for therapeutic angiogenesis in both myocardial and peripheral ischemia, these cells have not been systematically compared in clinical trials. In such studies there is also no apt means to discriminate the direct and indirect contribution of engrafted cells and it has generally been presumed, rather than established, that any vascular regeneration may be attributed to engrafted EPC.

Our aim in this study was to provide some basis to inform decisions on sourcing and possible *in vitro *manipulation of cells for vascular regeneration therapy. Unlike the use of HPC in hematopoietic reconstitution, where there is consensus that therapeutic efficacy can be related to the number of CD34^+ ^cells administered [16-18], there are no established *in vitro *tests to assess the relative angiogenic potency of a particular cell population. Very recent studies may have resolved issues over the phenotype of EPC that may allow their quantitation, but have yet to be widely accepted and applied [14]. In any case, vascular regeneration is likely to be complex and not simply dependent quantitatively on EPC availability, since there may be indirect paracrine potentiation of intrinsic angiogenic capacity by implanted cells as well as direct participation by incorporation of engrafted EPC [19-22,12]. We therefore adapted an established animal model of angiogenesis [23] to examine the effects of embedded test cells on the spontaneous vascularization of subcutaneously implanted sponge pellets in mice. Besides quantitative effects (vessel density in recovered sponges) this also allows vessel origin (mouse host or human implant) to be discriminated by species. Our aim in this investigation was to systematically evaluate and compare the effects in this model on vessel density and vessel origin of various potential clinical sources of EPC. A range of human sources of putative EPC and other cells of interest was embedded in sponge pellets prior to implantation, and recovered sponges were assessed both for changes in vessel density and for any incorporation of implanted human cells into vessel walls. In this study we have examined the *prima facie *differences between cells from different sources or enrichment/depletion from the same source, as they might be used clinically, in order to characterise these. We have not in this initial study attempted to investigate the underlying mechanisms behind the observed differences, which will be examined in subsequent studies, but we speculate on these by reference to the published work.

## Materials and methods

### Human cells

We prospectively examined the following potential sources of human EPC: 1) CD34^+ ^enriched and depleted MNC from different HPC sources; 2) putative proangiogenic monocytes; and 3) endothelial outgrowth cells (EOC) and mature human umbilical vein endothelial cells (HUVEC).

Samples were obtained, with appropriate informed consent under ethical approval granted by South East Scotland Research Ethics Committee 2 (reference 09/S1102/35), of human bone marrow aspirates, venous peripheral blood and umbilical cord blood, as previously described [24,25]. Mononuclear cells (MNC) were isolated by buoyant density centrifugation, HPC (CD34^+^) were enriched to > 95% purity using magnetic beads (MACS CD34 Isolation Kit, Miltenyi Biotec, UK) employing two passes on retention columns, and plastic (2-h) and fibronectin (48-h) adherent cells were enriched/depleted, all as previously described [24,25], where fibronectin adherence was carried out on fibronectin-coated vessels (BD BioCoat Cellware, Becton-Dickinson, UK) in EndoCult Medium (Stemcell Technologies SARL, Grenoble, France), and plastic adherence was carried out in polystyrene tissue culture flasks (Corning) in IMDM (Life Technologies, Scotland). In our hands 2-h plastic-adherent MNC or 48-h fibronectin non-adherent MNC both contain all the cells that give rise to CFU-Hill colonies, formerly called colony-forming units endothelial progenitor cells (CFU-EPC), after 4 to 6 further days culture on fibronectin in EndoCult Medium [24,25]. These are the so-called early EPC colonies now known to derive from monocytes [26-28]. As reported by others [29], we have found that these early CFU-EPC do not arise by proliferation: our time-lapse studies show that they arise by migration to aggregates, which subsequently disperse (unpublished). We cannot therefore enrich these cells by outgrowth culture and instead we selected MNC populations in which they reside. EOC and other outgrowth cells were obtained by outgrowth from MNC plated on type-1 collagen-coated polystyrene vessels (BD BioCoat Cellware, Becton-Dickinson, UK) in endothelial growth medium (EGM-2, Lonza) as previously described [25], after the method of Ingram *et al. *[30]. EOC colonies first appear at between 2 to 3 weeks in culture with frequent medium changes which remove non-adherent cells. EOC were derived from cord blood MNC, normal peripheral blood MNC and fetal liver MNC, but never from G-CSF-mobilized peripheral blood MNC [25]. Outgrowth cells obtained from bone marrow MNC by this method were consistently mesenchymal stromal cells (MSC), as reported by others [31]. These bone marrow derived MSC differentiated to bone or adipose tissue but failed to form tubules in Matrigel (Tura *et al*., manuscript submitted). HUVEC were purchased (cryopreserved Clonetics single donor cells, Lonza) and cultured in EGM-2 on uncoated tissue culture vessels (Corning). Unless otherwise stated, outgrowth cells and HUVEC were used after the second or third passage of expansion culture in EGM-2, where cells were passaged after reaching approximately 90% confluence. Where HUVEC isolates (not passaged) were used these were thawed on delivery and allowed to recover in culture for two days in EGM-2 before implantation.

### Sponge implant model

Male immunodeficient NSG mice aged 10 to 12 weeks were used in procedures approved by the University of Edinburgh ethics committee and authorized by the UK Home Office. Anesthetic comprised Vetalar (7.5 mg/ml) and Dormitor (100 μg/ml) in water for injection, given intraperitoneally at a dose of 0.065 μl/10 gbw. Reversal of anesthesia was induced, after at least 20 minutes of unconsciousness, using Antisedan (200 μg/ml) in water for injection. This was given subcutaneously at a dose of 0.05 ml/10 gbw. Mice were anesthetized and a sterilized polyurethane sponge cylinder (0.5 cm × 1 cm) (Caligen Foam, Accrington, Lancashire, UK) was implanted subcutaneously on each flank. Phenol-red free growth factor-reduced (gfr)-Matrigel^® ^(BD Biosciences) was used as vehicle to retain 1 × 10^5 ^test cells in sponges before implantation. Cells were spun down in tubes and resuspended in 100 μL ice-cold gfr-Matrigel, and a dry sterile sponge pellet was tamped into this in a tube to absorb the cell suspension (contralateral implant control sponges were loaded with gfr-Matrigel without cells). On coming to room temperature the Matrigel gelled, retaining the cells within sponges prior to implantation. We used gfr-Matrigel instead of normal Matrigel since gfr-Matrigel contains fewer growth factors, which might mask any cell-derived effects. There was no difference in spontaneous vascularization between untreated sponges (without gfr-Matrigel) and sponges with gfr-Matrigel (both without cells) in direct comparisons (paired in the same mice) in preliminary experiments. Replicate (groups of 4) mice were implanted simultaneously with each test cell isolate from any given donor. Each animal had a test sponge (gfr-Matrigel + cells) on one side and a control sponge (gfr-Matrigel alone) on the other side, as previously described [25]. Twenty-one days after implantation, mice were killed by asphyxiation in CO_2 _and sponges were excised. Sponges were fixed in 4% formalin and were embedded in paraffin wax for subsequent microtome sectioning for histological staining to quantify vessel density [23] and for immunohistochemical analysis to evaluate species origin.

### Histology and immunohistochemistry

Sponge sections (8 μm) were cut from paraffin blocks by microtome, mounted on glass slides, de-waxed, rehydrated and stained with hematoxylin/eosin for identification of blood vessels, characterised by a nucleated lumen surrounding non-nucleated eosin-stained red cells, as previously described [23]. Vessel density was determined by Chalkley counts as previously described [23], at three locations on each of two sections, with a microscope with a 25-point Chalkley eyepiece graticule and a ×40 objective.

Immunohistochemistry was developed for this study. Antibodies were pre-assessed for endothelial specificity and species cross-reactivity on sections from formalin-fixed and paraffin-embedded mouse and human vascular tissue, and on various cultured endothelial cells, using the same staining methods as used for experimental sponge sections. Sponge sections were stained with sets of paired anti-endothelial antibodies where rabbit cross-reactive (to mouse and human) antibody was paired with mouse human-specific antibody. Binding of rabbit and mouse antibodies was determined by subsequent staining with species-specific fluorochrome-conjugated secondary antibodies, namely goat anti-rabbit IgG Alexa 488 conjugate (green fluorescence) together with goat anti-mouse IgG Alexa 555 conjugate (red fluorescence). Diamidino-2-phenylindole (DAPI) staining (blue fluorescence) identified cell nuclei. The primary antibody combinations used were as follows: Set 1 (control): no primary antibodies (diluent only during first staining stage); Set 2: rabbit anti-α-smooth muscle actin (SMA) (Epitomics 1184-1) paired with mouse anti-CD31 (Dako M0823); Set 3: rabbit anti-CD31 (Epitomics 2540-1) paired with mouse anti-CD146 (AbCam ab49492); Set 4: rabbit anti-CD105 (endoglin) (Aviva ARP33069) paired with mouse anti-CD106 (VCAM-1) (Santa Cruz sc-13160); Set 5: rabbit anti-CD146 (Epitomics 2505-1) paired with mouse anti-vWf (von Willebrand Factor) (Monosan MONX10598).

Sections on microscope slides were de-waxed and rehydrated as for histological hematoxylin staining, then subjected to epitope recovery by simmering in EDTA solution pH 8.0 (Life Technologies) for 20 minutes in a microwave, then cooled and washed. These were blocked (Image-iT-FX signal enhancer, Life Technologies), then stained overnight at 4°C with one of a selection of cross-reactive rabbit anti-endothelial (monoclonal and polyclonal) antibodies mixed with one of a selection of human-specific mouse monoclonal antibodies, paired as above. Staining was in a diluent comprised of Dulbecco's phosphate buffered saline pH 7.2 containing 0.5% Tween 20, 0.01% sodium azide and 2% goat serum and washes used the above diluent without goat serum. Primary and secondary antibodies were used generally at 1/200 in the diluent, and staining was carried out on duplicate sections mounted on each slide, using 150 μL of diluted antibodies on slides mounted, blocked and pre-washed in a staining rack (Shandon Sequenza). Sets of sections from one or more sponges were stained simultaneously with the panel of different primary antibodies above. After multiple washes, binding of rabbit and mouse antibodies was determined by subsequent staining for 3 h at 37°C with a mixture of secondary antibodies goat anti-mouse IgG Alexa 555 conjugate (red fluorescence) (Life Technologies A21424) and goat anti-rabbit IgG Alexa 488 conjugate (green fluorescence) (Life Technologies A11034), followed by washing then mounting under coverslips in anti-fade mountant (ProLong Gold, Life Technologies) containing DAPI (blue fluorescence) to identify cell nuclei. Stained and mounted slides were stored at 4°C in the dark.

For each primary antibody pair two sections from each sponge were stained, and were examined using a Zeiss Axio-Observer A1 microscope equipped with appropriate filter combinations, and illustrative images were recorded using a high sensitivity Zeiss AxioCam MRm (black & white) camera. For each field images were captured under normal tungsten (phase-contrast) illumination and under UV illumination for each of blue, green and red channels with appropriate filters for each fluorochrome. Automated exposure for image capture (determined by Zeiss AxioVision software V 4.8) was optimized on the brightest object in a given field (max/min exposure). Images were captured, colored according to channel, and processed for single and combined channel images using Zeiss AxioVision (v 4.8) software. Detection of green fluorescence was taken as indicating binding of the primary rabbit cross-reactive antibody, and detection of red fluorescence was taken as indicating binding of the primary mouse human-specific antibody. Presence of green without red indicated mouse (host) tissue while both green and red together indicated human (cell implant) tissue: nucleated cells stained with DAPI (blue). Erythrocytes within perfused vessels were autofluorescent in both the red and green channels and lacked nuclei by DAPI stain. In the absence of specific immunofluorescence, erythrocyte autofluorescence dominated captured images due to longer automated exposures, but could appear minimal in any channel that showed bright specific immunofluorescence, which was maximal at shorter exposures. In the presence of immunofluorescence in only one (green or red) channel, the erythrocyte autofluorescence image was diminished in that channel but retained in the other, so that mouse-only vessels showed green immunofluorescent walls (from cross-reactive rabbit anti-endothelial antibody) surrounding red autofluorescent erythrocytes. Primary interpretation was based on extensive visual examination using a ×40 objective of the whole sponge section, and on comparison to control unstained sections (without primary antibodies) and to host (mouse-only) vessels in sections from spontaneously vascularized contralateral-control sponges without implanted human cells. Identification of integration of human cells into vessels was based on visual scanning for red fluorescence resulting from bound human-specific antibody. Representative images of selected fields were recorded for illustration, and after processing provided composite multicolor images showing co-localisation of specific immunostaining and relative location of autofluorescence. Patterns of specific immunofluorescence and the relative brightness of erythrocyte autofluorescence in images also informed interpretation of whether or not implanted human derived cells were incorporated into the sponge vessels.

### Statistical methods

Results are reported for equivalent cell fractions from different donors (different mouse group replicates) which were pooled and analyzed by repeat-sample analysis of variance (ANOVA) as in our previously described use of the sponge implant model [23], using NCSS 2007 software (Statistical Solutions).

## Results

### Implantation of CD34+-enriched or -depleted mononuclear cells

**Figure 1:**
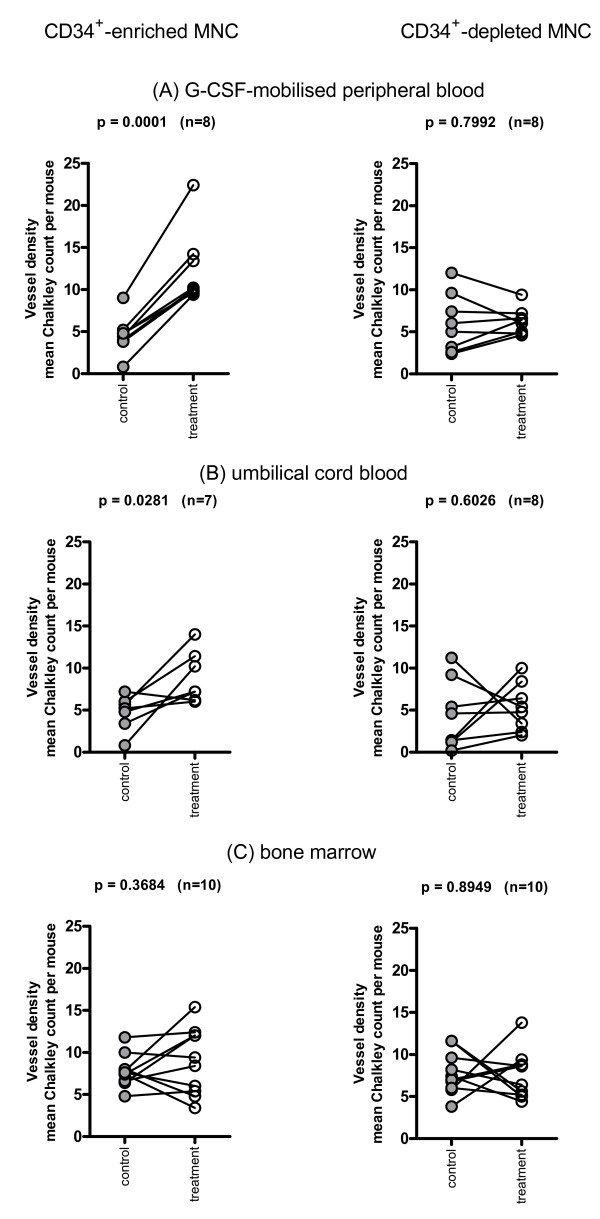
**CD34+-enriched mononuclear cells increase angiogenesis in sponges**. Impregnation of sponges with CD34+-enriched MNC (mononuclear cells) from **(A) **granulocyte colony-stimulating factor (G-CSF)-mobilized peripheral blood or **(B) **umbilical cord blood, significantly increased vascular density within sponges compared to contralateral sponges without cells. CD34+-enriched MNC from bone marrow **(C) **and CD34+-depleted cells from any source had no effect. The replicate means of paired Chalkley counts are shown for all mice receiving each kind of cell fraction from each source. The data are pooled from different experiments where different donors contributed to sets of enriched and depleted cells. Data were analyzed comparing individual count results for sponge pairs from each mouse (*n *= number of mice) for each cell type and source by repeat-measure analysis of variance.

**Figure 2:**
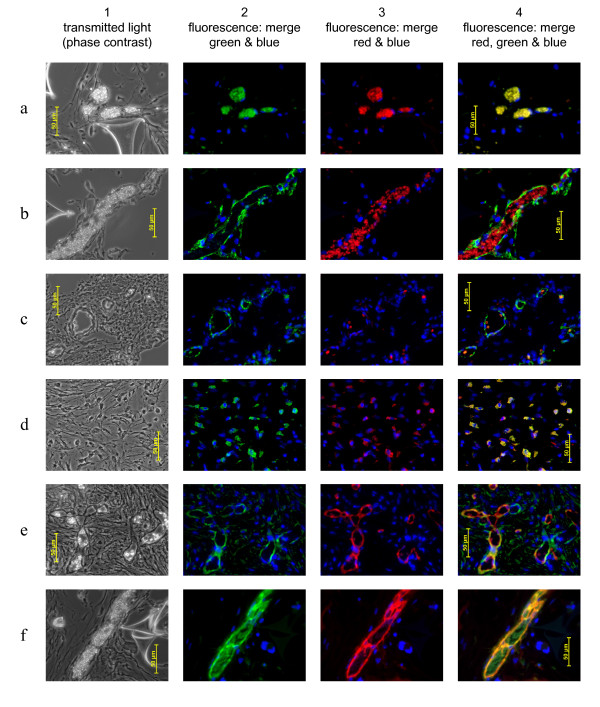
**Representative examples of vessels detected in sponge sections**. The images show **(a**) unstained control; **(b, c) **mouse-only vessels; **(d) **free human cells not in vessels; and **(e, f) **vessels with human cells in vessel walls. Cells implanted: **(a) **endothelial outgrowth cells (EOC); **(b) **CD34+-enriched cord blood MNC; **(c**) CD34+-depleted cord blood MNC; **(d) **plastic-adherent MNC (> 80% monocytes); **(e) **EOC; **(f) **EOC. Column 1 is the phase-contrast image; column 2 is the image from the green fluorescence channel merged with blue fluorescence showing diamidino-2-phenylindole (DAPI) nuclear stain; column 3 is red fluorescence merged with the blue DAPI nuclear stain; and column 4 is the merged image from green, red and blue fluorescence. The antibody pairs shown are **(b, e) **cross-reactive (rabbit) anti-α-smooth muscle actin with human-specific (mouse) anti-CD31 and **(c, d, f) **cross-reactive (rabbit) anti-CD31 with human-specific (mouse) anti-CD146. Human cells are identified by red fluorescence revealing bound human-specific antibody **(d, e, f)**, which generally co-localizes with the green fluorescence of bound cross-reactive antibody. Where only cross-reactive antibodies bind **(b, c)**, the vessel walls show green fluorescence only, indicating mouse tissue only. In one example **(e) **some mouse-only (green without red) vessels can be seen together with human vessels (green and red). Where no antibodies bind, as in the control **(a)**, both red and green erythrocyte autofluorescence is evident in the images, but only the red erythrocyte autofluorescence is evident **(b, c) **when vessel walls show green immunofluorescence, and little or no erythrocyte autofluorescence is evident when vessel walls show both red and green fluorescence. A more comprehensive set of images is given in Additional file 1.

MNC from G-CSF-mobilized peripheral blood, umbilical cord blood and bone marrow and their CD34-enriched and -depleted fractions were assessed in a series of experiments (Table 1). Unfractionated MNC from any source had no apparent effect on vessels (not shown). CD34-enriched MNC from G-CSF-mobilized peripheral blood (Figure 1A) or umbilical cord blood (Figure 1B) significantly increased vessel density, whereas the corresponding CD34-depleted MNC from any source had no effect on vessel density (Figure 1). CD34-enriched MNC from bone marrow had no effect on vessel density (Figure 1C). Immunohistochemical analysis showing representative examples of vessels comprised exclusively of mouse cells is shown in Figure 2 where (b) CD34-enriched cord blood MNC or (c) CD34-depleted cord blood MNC were implanted in sponges. Further examples of vessel immunohistochemistry images using sponges with a range of implanted cells and stained with the different primary antibody combinations are shown in Additional file 1. None of these implanted MNC from any source, or whether the MNC were CD34-enriched or depleted, incorporated into vessels (Table 1).

**Table 1:** Implantation of CD34+-enriched mononuclear cells from mobilized blood or cord blood enhances vascularization in subcutaneous sponge implants without incorporation into vessels

		Vessel density (Chalkley counts)				
**Source**	**Implanted cells**	**Untreated**	**Treated**	**Difference**	**Probability level (ANOVA)**	**Incorporation into vessels**

Mobilized peripheral blood (donors: *n *= 2)	CD34-enriched MNC (mice: *n *= 8)	3.833 (0.661)	10.250 (1.254)	6.417 (0.785)	0.00008*	No
	CD34-depleted MNC (mice: *n *= 8)	5.021 (1.023)	5.208 (0.465)	0.187 (0.710)	0.799	No
						
Umbilical cord blood (donors: *n *= 2)	CD34-enriched MNC (mice: *n *= 7)	3.929 (0.655)	7.405 (0.957)	3.476 (1.207)	0.028*	No
	CD34-depleted MNC (mice: *n *= 8)	3.604 (1.200)	4.437 (0.836)	0.833 (1.568)	0.603	No
						
Bone marrow (donors: *n *= 3)	CD34-enriched MNC (mice: *n *= 10)	6.483 (0.509)	7.433 (1.047)	0.950 (1.003)	0.368	No
	CD34-depleted MNC (mice: *n *= 10)		6.317 (0.752)	-0.167 (1.226)	0.895	No

Implantation of CD34^+^-enriched mononuclear cells (MNC) from granulocyte colony-stimulating factor (G-CSF)-mobilized peripheral blood or umbilical cord blood significantly increased new vessel formation in subcutaneous sponges (*P < 0.05). Vessel density was increased between paired cell-impregnated sponges compared to contralateral control sponges without cells. Implantation of CD34^+^-enriched MNC from bone marrow and CD34^+^-depleted MNC from any source had no significant effect on vessel density. No human cells incorporated into vessels. Data are means, with standard errors in parentheses. Probability was determined by repeat-sample analysis of variance (ANOVA) on individual readings for each mouse from pooled data from different donors.

### Implantation of putative proangiogenic monocytes

Neither plastic-adherent MNC, their residual non-adherent counterparts, nor MNC not adherent to fibronectin after 48-h culture had any effect on endogenous vessel density, nor did they incorporate into vessels (Table 2). All vessels stained with the cross-reactive rabbit anti-endothelial cell antibodies but none stained with human-specific mouse anti-endothelial antibodies, indicating these vessels were comprised exclusively of endogenous mouse cells. However, when plastic-adherent MNC (> 80% monocytes) were implanted, some microscopy fields contained dispersed cells (not in vessels) of human origin since they stained with the human-specific antibodies as well as the cross-reactive antibodies (Figure 2d). These dispersed cells were also detected less prominently in other experiments when MNC containing monocytes were implanted. It was noted that these isolated cells were also detected by anti-CD105 (endoglin) paired with anti-CD106 (see Additional file 1, Figure 4b). The anti-CD106 does not detect human endothelial cells which have integrated into vessels (see below). These cells were interpreted as human monocytes that have upregulated expression of endothelial markers (CD31, CD105, CD106, CD146) during the 3 weeks of implantation. They were not apparently detected (none were seen) by anti-α-SMA or anti-vWf antibodies.

**Table 2:** Implantation of putative proangiogenic monocytes does not stimulate vascularization in subcutaneous sponge implants

		Vessel density (Chalkley counts)				
**Source**	**Implanted cells**	**Untreated**	**Treated**	**Difference**	**Probability level (ANOVA)**	**Incorporation into vessels**

normal peripheral blood (donors: *n *= 3)	Adherent (plastic) MNC (mice: *n *= 11)	5.288 (0.742)	5.106 (0.590)	-0.182 (0.796)	0.475	No
	Non-adherent (plastic) MNC (mice: *n *= 12)	5.806 (0.844)	5.514 (0.840)	-0292 (1.116)	0.799	No
						
mobilized peripheral blood (donors: *n *= 1)	Adherent (plastic) MNC (mice: *n *= 4)	4.833 (0.791)	6.083 (0.647)	1.25 (0.610)	0.133	No
	Non-adherent (plastic) MNC (mice: *n *= 4)	6.208 (2.197)	7.583 (1.674)	1.375 (3.152)	0.692	No
						
normal peripheral blood (donors: *n *= 1)	Non-adherent (fibronectin) MNC (mice: *n *= 4)	2.667 (0.853)	4.333 (1.080)	1.667 (1.393)	0.318	No

Implantation of putative proangiogenic monocytes selected on the basis of mononuclear cells (MCN), which contain the cells responsible for early colony-forming units endothelial progenitor cells (CFU-EPC) by rapid (2h) adherence to plastic or non-adherence to fibronectin after 48 h [24,25], did not significantly increase new vessel formation in subcutaneous sponges (*P *> 0.05 for all). Vessel density was not different comparing paired cell-impregnated sponges to contralateral control sponges without cells. No human cells incorporated into vessels. Data are means, with standard errors in parentheses. Probability was determined by repeat-sample analysis of variance (ANOVA) on individual readings for each mouse from pooled data from different donors.

### Implantation of endothelial and other outgrowth cells

EOC were cultured, as previously described [25], from umbilical cord blood and normal peripheral blood, and were also obtained from fetal liver MNC. EOC could not be obtained from G-CSF-mobilized peripheral blood by this (or any other) method, as previously reported [25], so were not available for implantation. EOC from all sources were incorporated into the newly formed vessels in sponges (Table 3). In these vessels, immunoreactivity was detected as homogeneous luminal binding of human-specific mouse anti-CD31 (Figure 2e) and anti-CD146 (Figure 2f) which appeared to co-locate with cross-reactive anti-α-SMA and cross-reactive anti-CD31 binding, respectively, and also punctate luminal binding of anti-vWf co-located with homogeneous cross-reactive anti-CD146 binding (see also Additional file 1, Figures 2b, 3b and 5b), demonstrating the presence of human endothelial cells. Human CD106 was not detected on vessels in sponges in which human vessel markers were detected extensively by other antibodies, but human CD106 was detected in other sponges on isolated monocyte-derived human cells not in vessels (see above). From this we infer that CD106 is not expressed on human endothelial cells that have integrated into the neo-vessels. Since we could not specifically detect the vessels containing human cells with anti-CD106, we could not assess their co-labeling with the paired cross-reactive anti-CD105 (endoglin), which diffusely labeled vessels both in the lumen (Additional file 1, Figure 4a) and apparently in some cases, also in perivascular cells (Additional file 1, Figure 4a, row C). Where vessels containing incorporated human cells were identified in sponge sections, they tended to occur in localized areas and not throughout the sponge section, where in general the majority of vessels were of host origin and contained only mouse endothelial cells. All vessels contained mouse erythrocytes indicating that they were perfused and contiguous with the host circulation. Unlike mobilized blood and cord blood CD34^+ ^MNC (Table 1), these cells did not enhance endogenous vascularization in the sponges despite their incorporation, since there was no increase in vessel density compared to control paired contralateral sponges without implanted cells.

**Table 3:** Implantation of EOC, HUVEC or MSC does not enhance vascularization, but EOC incorporate into vessels

		Vessel density (Chalkley counts)				
**Source**	**Implanted cells**	**Untreated**	**Treated**	**Difference**	**Probability level (ANOVA)**	**Incorporation into vessels**

Umbilical cord blood or normal peripheral blood (donors: *n *= 5)	Endothelial outgrowth cells (mice: *n *= 20)	7.528 (0.581)	9.403 (0.671)	1.875 (0.967)	0.078	Yes
Umbilical vein wall (donors: *n *= 2)	Human umbilical vein endothelial cells (mice: *n *= 7)	8.238 (1.553)	8.833 (1.492)	0.595 (1.502)	0.706	No
Outgrowth from foetal liver MNC (donors: *n *= 1)	Endothelial outgrowth cells) (mice: *n *= 4)	9.25 (1.975)	9.25 (0.308)	0.00 (2.174)	1.000	Yes
Outgrowth from bone marrow MNC ***(donors: n = 1)***	Mesenchymal stromal cells (MSC) ***(mice: n = 4)***	5.958 (0.893)	8.583 (1.788)	2.625 (2.260)	0.329	No
						
Umbilical cord blood (donors: *n *= 1)	EOC - extended culture (11**^th ^**passage) (mice: *n *= 2)	3.667 (2.000)	9.333 (2.500)	5.667 (0.500)	0.056	No
Umbilical vein wall (donors: *n *= 1)	HUVEC - early culture (pre first passage) (mice: *n *= 4)	3.958 (1.214)	5.167 (0.649)	1.208 (1.102)	0.353	Yes

Implantation of endothelial outgrowth cells (EOC), human umbilical vein endothelial cells (HUVEC) or mesechymal stromal cells (MSC) did not significantly increase new vessel formation in subcutaneous sponges (*P *> 0.05), as measured by the difference in vessel density between paired treated sponges containing implanted cells and contralateral control sponges without cells. Vessel density was not different comparing paired cell-impregnated sponges to contralateral control sponges without cells. EOC clearly incorporated into newly formed vessels in all sponges examined, whereas neither HUVEC nor MSC incorporated. Exceptions were late-passage EOC which did not incorporate, or very early HUVEC which did incorporate. Data are means, with standard errors in parentheses. Probability was determined by repeat-sample analysis of variance (ANOVA) on individual readings for each mouse from pooled data from different donors.

Under EOC culture conditions, MNC from bone marrow consistently produced mesenchymal stromal cells (MSC), not EOC, as has been reported by others [31]. These MSC had a distinct phenotype from EOC, and unlike EOC could differentiate to osteoblasts or adipocytes but could not produce endothelial tubules in Matrigel^® ^(Tura *et al.*, manuscript submitted). These MSC did not enhance vascularization in sponges or incorporate into vessels (Table 3). Effectively, of all the human cells tested (Tables 1, 2 and 3) only EOC show consistent incorporation into vessels. In contrast to EOC, HUVEC showed no incorporation into vessels when EOC or HUVEC were obtained by our routine culture of two to three passages from first confluent outgrowth. However, EOC were unexpectedly found to lose the ability to incorporate into vessels after extensive culture, in this case 11 passages (Table 3). In response to this result, and by way of contrast, an early HUVEC isolate (not grown to confluence or passaged) was examined, and was found to still express the ability to incorporate into vessels (Table 3 and Additional file 1, Figure 3b, row B). HUVEC did not enhance vascularization in sponges (Table 3).

In our hands, in direct comparisons, cord blood MNC produced EOC only on culture vessels coated with type-1 collagen (or sporadically on gelatin, which contains type-1 collagen), and the same MNC did not produce EOC when cultured on type-IV collagen, fibronectin or uncoated tissue culture polystyrene. This was confirmed in three different cord blood donors.

## Discussion

These results reveal that angiogenic potency is not simply dependent proportionally on EPC numbers, as proposed in many prevailing interpretations, but is complex and may involve multiple cell types with different roles, as has been reviewed [11,12]. In our model the angiogenic potency appears to comprise two distinct activities mediated by different cells, 1) true endothelial progenitor activity with cell incorporation into vessel walls provided by EOC, and 2) proangiogenic amplification of vascularization provided by CD34^+ ^HPC from mobilized peripheral blood and cord blood, but not from bone marrow. Different sources of HPC that may have essentially similar clinical hematopoietic potency can have quite different angiogenic properties, and HPC content does not predict angiogenic potential. Other cells, such as monocytes or mesenchymal stromal cells, seem irrelevant in this model.

This distinction was not apparent when we proposed this study in 2006 in anticipation of a need to supply cells for therapeutic vascular repair in myocardial infarction or severe peripheral ischemia, which was and still is focussed primarily on the use of autologous HPC from bone marrow or mobilized blood, with or without enrichment of HPC by selection of CD34^+ ^or CD133^+ ^cells. During our studies a number of reports have appeared, which show 1) that HPC have a proangiogenic effect, particularly CD133^+ ^HPC [19]; 2) that EOC probably represent true endothelial progenitors that incorporate into neo-vessels and are derived from CD34^+^CD133^- ^cells and not CD34^+^CD133^+ ^cells [13,15]; 3) that early CFU-EPC are derived from monocytes and while these may express some endothelial markers, they are unrelated to EPC, do not proliferate, and do not incorporate into vessels [26-28]; and 4) that mesenchymal stromal cells do not contribute directly to angiogenesis but may support it [32]. It is possible to review these individual reports and come to essentially the same conclusion that we do from our study, so our conclusions are not original. However, papers contradicting these findings continue to be published, so it is important to provide support for these conclusions. Also, instead of focussing on a particular cell, our work systematically compares under the same conditions, a number of different proposed sources and subpopulations of cells for therapeutic vascular repair and shows that they are not equivalent. We provide important support for the various individual studies above, for which there is not yet a consensus to judge from the range of interpretation and opinion in many current publications, and may help provide support for informed redesign of cellular therapy for vascular repair.

The sponge model, in which the implanted sponge is spontaneously encapsulated and vascularized by the host, is often called an inflammatory angiogenesis model [33]. It is an established, widely used model which allows investigation of angiogenesis *in vivo *and which was well suited for the comparison of the effects of different implanted cells on new vessel formation [23,34]. It is unlikely that the model replicates exactly the process of reparative angiogenesis in ischemic tissue [34], and these results may not necessarily be extrapolated to pathophysiological angiogenesis (for example, in response to myocardial infarction or in limb ischemia). In our hands the sponge model adapted well for the comparison of the effects of different implanted cells on the angiogenesis that occurs within these sponges, where it discriminates between the direct incorporation of implanted cells into neo-vessels and the proangiogenic stimulation of vessel number by different implanted cells. However, it may be that more subtle effects could be masked by the inflammatory response to the sponges [33].

In our experience, EOC derive only from a subset of CD34^+^/CD133^- ^MNC[25], confirming previous reports [13,15]. This is contrary to a number of publications stating that CD133 is found on EPC. This inconsistency in definition confounds an objective assessment of many published reports of relationships between EPC and angiogenic potential that employ different phenotype bases to quantify or enrich EOC, as reviewed recently [14]. The subset of CD34^+^/CD133^- ^cells recently described by Estes *et al. *[35] as defining the true EPC phenotype are extremely rare in circulation and also rare in accessible sources of HPC such as umbilical cord blood or mobilized peripheral blood. We find a similar very rare (fewer than one in a million) MNC subpopulation giving rise to EPC that are CD34^+^/CD133^-^/CD45^-^/CD146^+^. If CD146 is an alternative to CD31 for discriminating EPC then these cells may be the same CD34^+^/CD133^-^/CD45^-^/CD31^+ ^subpopulation described by Estes *et al. *[35] as EPC with *in vivo *vessel-forming capacity. These CD34^+^/CD133^-^/CD45^-^/CD146^+ ^cells can be detected in normal peripheral blood and cord blood MNC, both of which give rise to EOC, but are below our limit of detection in bone marrow or mobilized blood MNC, neither of which give rise to EOC (Tura *et al.*, manuscript submitted). Thus, detection of this cell subpopulation appears to coincide with our ability to grow EOC from these different sources. We find from outgrowth studies that the frequency of EOC colonies (endothelial colony-forming cells, ECFC) in cord blood MNC is approximately one in five million, and is approximately one in thirty million in normal peripheral blood MNC. ECFC frequency remains low even in CD34-enriched MNC, indicating that EPC are a very small component of total CD34^+ ^cells, but no ECFC are found in CD34-depleted MNC [25]. Therefore, apart from homogeneous EOC from culture, the cell populations implanted in this study will contain very few or no true EPC as indicated by the very low ECFC frequency, even when CD34^+^-enriched cord blood cells are used [25]. It is not surprising, therefore, that no human cell incorporation into sponge vessels was found in these cases.

It may be that adult EPC have been ascribed a close relationship to HPC by their coincidental expression of CD34, which is a marker of both hematopoietic progenitors and mature endothelial cells. However, HPC and EPC might not be as closely related in adults as inferred, and circulating EPC might not derive from bone marrow. The endothelial stem cell niche could be principally located elsewhere, such as in the vasculature itself. Ingram *et al. *[36] have described a hierarchy of endothelial lineage cells, including progenitors that can be recovered from vessel walls. This may be why we consistently fail to recover EOC from G-CSF mobilized blood MNC, where HPC leave the bone marrow and appear in the circulation but are not apparently accompanied by EPC, which give rise to EOC [25]. Furthermore, existing circulating EPC could be diluted to below detection by the leukocytosis which accompanies G-CSF mobilization. This could also explain why we and others [31] were unable to grow EOC from bone marrow MNC and instead obtain mesenchymal stromal cell outgrowth under conditions that generate EOC when other cell sources are used. These bone marrow-derived MSC had no effect in our sponge vascularization model, showing neither incorporation into vessels, nor promotion of vascularization. Similarly, purchased MSC derived from human adipose tissue had no effect on sponge vascularization (not shown).

Melero-Martin *et al. *[32] have shown that implanted MSC can contribute to vascularization in implanted Matrigel plugs by synergizing with implanted human EOC in co-transplants. Here it was proposed that the MSC may contribute by secretion of vascular endothelial growth factor (VEGF) to induce implanted human EOC to form vessels, and the MSC may also stabilize vessels by forming perivascular structure around the endothelial-derived lumen. However, they found that EOC implanted alone did not result in vascularization of the Matrigel plug and neither is it spontaneously vascularized by the host. In contrast in our sponge implant model sponges are spontaneously vascularized by the host, and MSC implant did not apparently affect this spontaneous vascularization whereas mobilized or cord blood CD34^+ ^cells significantly promoted it. From this we can infer that these CD34^+ ^cells are more potent than MSC in promoting angiogenesis. We did not study co-implantation of different cell types, but human EOC implanted alone without accompanying MSC incorporated into growing vessels while MSC alone did not, and neither EOC nor MSC promoted increased vessel density. It seems that the sponge model and the Matrigel plug model differ. The sponge model has been used extensively as a model of angiogenesis and to measure effects of exogenous factors on this angiogenesis [37,23,34,25]. It may be that since no spontaneous host vascularization takes place in the Matrigel plug, that model may reflect *de novo *vasculogenesis. It may also be that vasculogenesis and angiogenesis depend differently on EPC and accessory cells, accounting for the differences between our findings and those reported by Melero-Martin *et al. *[32]. Since the sponge model indicates that available EOC are incorporated when new vessels form, it may be that the Matrigel plug model says more about the ability of the cells that are co-transplanted with EOC to promote vessel formation than about the inherent capacity of EOC to form vessels.

Melero-Martin *et al. *[32] also used anti-α-SMA antibody to discriminate MSC and perivascular cells from luminal endothelial cells. In our studies we employed cross-reactive anti-α-SMA which did not discriminate mouse (host) from human (implant) cells. This antibody stained brightly and in most cases it appeared to stain the endothelial lumen of vessels (Figure 2 and Additional file 1, Figures 2a and 2b) as has been reported for some human and mouse vessels [38]. It also stained human EOC and HUVEC grown *in vitro *(not shown), which have also been reported to express α-SMA[39], so anti-α-SMA is not specific for perivascular cells and is expressed on luminal endothelial cells, at least in some cases. In some areas of most sponge sections acellular (non-nucleated) deposits of α-SMA were identified by this antibody (Additional file 1, Figure 2a, row B and Figure 2b, row C). This anti-α-SMA also stained perivascular cells in some vessels (see Additional file 1, Figure 2a, rows A and C) in that such cells appeared in an adjacent layer peripheral to the lumen, and often with their long axis vertical to the lumen. Similarly, apparent perivascular layers peripheral to the lumen in mouse-only vessels were also identified with anti-CD31 (Additional file 1, Figure 3a, row A), anti-endoglin (CD105) (Additional file 1, Figure 4a row C) and anti-CD146 (Additional file 1, Figure 5a, row B). Thus, all the cross-reactive antibodies, including anti-α-SMA, did not discriminate between endothelial or perivascular cells. This also differs from the report by Melero-Martin *et al. *[32], where α-SMA is claimed to be associated only with MSC and not endothelial cells. The occurrence of vessels with perivascular layers was confined to a minority of mouse-only vessels, was never found where human cells incorporated into vessels, and had no discernible association with any implanted cell type. We draw no conclusions from this but note their presence and their prominent vivid staining, especially by anti-α-SMA, but also by other antibodies.

While HUVEC, expanded routinely *in vitro *through two to three passages, showed normal growth and tubule formation in Matrigel, they did not incorporate into vessels when implanted in sponges. In contrast, freshly isolated HUVEC, not yet passaged, incorporated into host vessels (Table 3). According to Ingram *et al.*, freshly isolated vessel endothelial cells should contain EPC [36], but it appears that they quickly lose the ability to incorporate into vessels on expansion *in vitro*. EOC also eventually lost the ability to incorporate after extensive passage (Table 3). We have not characterized the kinetics of this loss of incorporation ability either in EOC or HUVEC, but it could reflect a change from progenitor to mature endothelial cell. We have not identified anything within growth characteristics, expression of a wide range of surface markers, or patterns of expression of various genes, that discriminates EOC from mature HUVEC other than this ability to incorporate into vessels in the sponge model of angiogenesis (Tura *et al.*, manuscript submitted). This should be examined further. We also note that while EOC were cultured on type-1 collagen, HUVEC were cultured on uncoated plastic, and the possible role of extracellular matrix in maintaining immature status or promoting maturation should be investigated. Cells for therapeutic use should be capable of incorporation into vessels, so it is important to retain this property during *in vitro *expansion if EOC are to be used for vascular regenerative therapy. The sponge model can examine this property.

Neither adherent monocytes, nor MNC cultured on fibronectin, influenced sponge vascularization by incorporation or proangiogenic activity. Indeed, of all the cells studied, adherent monocytes slightly, but not significantly, inhibited vascularization (Table 2). However, isolated human cells expressing endothelial markers were found in the sponges in which monocyte-enriched cells were implanted. The derivation of early CFU-EPC from monocytes on fibronectin, and numerous other reports of expression of endothelial markers by monocytes when cultured on fibronectin [26,27,29], suggest that the cells observed in the sponges are monocytes, or were derived from them. The culture of MNC on either fibronectin or collagen to produce putative EPC has caused some confusion between true EOC and differentiated monocytes. Our experience (unpublished) suggests that culture on fibronectin produces monocyte-derived cells that can mimic endothelial cells by phenotype, whereas, consistent with other reports [28], true EPC are produced only on collagen type-1. However, others have described production of functional EOC on fibronectin [40] or even on uncoated plastic[41] under certain circumstances, so it may be that the collagen effect is quantitative rather than mandatory. However, since monocytes appear to require fibronectin for endothelial-like differentiation and formation of early CFU-EPC, the exclusive use of type-1 collagen for endothelial outgrowth from MNC might avoid any confusion between true EOC and differentiated monocytes. Since monocytes did not apparently influence vessel growth in this study, they may not be an important component of cellular therapy for vascular regeneration, and may be irrelevant when considering acquisition of cells for that purpose. However, since there is a non-specific host inflammatory response to the sponge and migration of host inflammatory cells, including monocytes, into the sponge, it may be that this masks any stimulatory effect provided by the addition of human monocytes. If monocytes or their derivatives have a masked proangiogenic effect, it must be different from the proangiogenic effect of HPC, which is evident. It has also been suggested that monocytes may have an ability to infiltrate ischemic tissue and provide pathways for vascularization by endothelial cells [42,43], which may not have been tested in this model.

While EOC have been proposed as an option for therapeutic vascular regeneration [44,41], to date all clinical trials have employed HPC from patients' autologous bone marrow or G-CSF-mobilized peripheral blood. Some recent studies show that bone marrow-derived HPC-like cells do not contribute to angiogenesis by incorporation into regenerating vascular endothelium [45-47]. Our study shows that the prominent, and possibly the only, angiogenic potential of HPC-like cells is proangiogenic activity, not as sources of EPC. This proangiogenic activity is not seen at low HPC frequency in unfractionated MNC and is only found when HPC are enriched through CD34^+ ^cell selection, so is evidently quantitatively related to CD34^+ ^cell numbers. Neither EOC nor HUVEC showed a proangiogenic effect despite EOC and early HUVEC showing incorporation into vessels, so their role seems passive, dependent only on availability. It is notable that the strongest, most significant proangiogenic effect came from G-CSF-mobilized CD34^+ ^cells, followed by cord blood CD34^+ ^cells, and that surprisingly bone marrow-derived CD34^+ ^cells were not proangiogenic in this model. While HPC may be characterized by expression of CD34 or CD133, these markers are not equivalent or homogeneously expressed by HPC, and different subpopulations exist which are CD34^+^CD133^-^, CD34^+^CD133^+ ^and CD34^-^CD133^+ ^[24]. The observed proangiogenic capacity follows the proportions that we have found in different HPC sources of CD34^+ ^cells that co-express CD133, which are greatest in mobilized blood HPC (around 80%), intermediate in cord blood HPC (around 53%) and smallest in bone marrow HPC (around 13%)[24]. It has been reported that CD133^+ ^HPC are proangiogenic, without incorporating into neovessels [19,48]. Recently it has been shown that in subpopulations of CD34^+ ^HPC that differ in complex phenotype only by CD133 expression, the CD133^+ ^cells show proangiogenic activity, while the CD133^- ^cells do not [49]. Thus, it may be that the proangiogenic effect is mediated only by (some or all) CD133^+ ^cells, but not by CD133^- ^cells. This might explain the differences we found between the various sources of HPC, reflecting the proportions of CD34^+ ^cells that co-express CD133^+^. If so, this could indicate that expression of CD133 may be better than expression of CD34 for selection for proangiogenic HPC. Further studies are needed to determine and characterize subpopulations of HPC-expressing proangiogenic activity, and whether they can be expanded or enhanced *in vitro *for therapeutic use.

This study suggests that any observed clinical benefit reported in trials of HPC therapy for vascular regeneration has predominantly been mediated by indirect proangiogenic effects rather than through direct incorporation of any EPC contained within these HPC sources. This may especially be the case in trials that have employed CD133^+ ^cells enriched from HPC sources, since these would be depleted of CD34^+^CD133^- ^cells recognised as EPC. Since the main therapeutic targets remain myocardial and severe peripheral ischemia, and the therapeutic options are confined to use of autologous cells because of requirements for histocompatibility, the current clinically available HPC sources are either bone marrow or mobilized peripheral blood HPC. Neither of these contain EPC detectable through endothelial outgrowth, and their CD34^+ ^cells appear at opposite ends of the spectrum in their proangiogenic activity. Since neither source seems to provide EPC, it seems from this study that since mobilized blood HPC are the more potent proangiogenic cells, they could be preferred over bone marrow for autologous therapeutic vascular regeneration. With regard to provision of EPC for therapeutic use, the best available source for autologous EOC would seem to be normal peripheral blood, not bone marrow or mobilized peripheral blood HPC. It should be possible to produce autologous EOC for therapeutic use [41,50], and clinical trials are needed to evaluate the effect of EPC distinct from or in synergy with the proangiogenic effects of HPC therapies. Since the endothelial progenitor cell has been the primary focus of most studies, new studies are now required to clarify the identity of the proangiogenic effector cell and the mechanisms by which it delivers its effect, which may have been the only clinical effect observed until now. It may be that since the proangiogenic cell does not integrate into tissue, it may have less stringent histocompatibility requirements for clinical use, or the proangiogenic effect could be delivered by cell-free systems. The precursor and proangiogenic activities appear to be the main components required to improve the design of cellular therapy for vascular regeneration. The role of other cells such as MSC or monocytes may be relatively marginal, but might provide some added benefit once the main progenitor and proangiogenic components are optimized.

## Conclusions

Clinical vascular regeneration trials employing autologous hematopoietic progenitor cells have been based on the assumption that endothelial progenitors coexist with hematopoietic progenitors, and may share a common precursor. It is further assumed that endothelial progenitors home to sites of vascular regeneration and contribute to vessel formation by incorporating into new vessels. These assumptions are complicated by the fact that distinct endothelial and hematopoietic progenitors share many phenotypic markers, and that hematopoietic progenitors can promote angiogenesis. This study of the effects of different human cells on spontaneous vascularization in a mouse model supports recent studies showing that the contribution of autologous HPC to vascular regeneration comes from indirect proangiogenic effects. EPC that give rise to EOC, which incorporate into newly formed vasculature, are extremely rare in HPC sources. EOC could be isolated only from normal peripheral blood or umbilical cord blood, but could not be isolated from bone marrow or G-CSF-mobilized peripheral blood, which have until now been the sources of autologous HPC used for therapeutic vascular regeneration. Further, mobilized peripheral blood CD34^+ ^cells (HPC) were the most potent proangiogenic cells, and bone marrow CD34^+ ^cells were surprisingly without proangiogenic activity at the cell doses tested. This may indicate that G-CSF-mobilized peripheral blood may be preferred to bone marrow for cell therapy for promotion of vascular repair. Further studies are needed to determine whether true EPC have a role in therapeutic vascular repair, and to elucidate the nature of the differences in proangiogenic potency between different HPC sources of CD34^+ ^cells.

## Abbreviations

ANOVA: analysis of variance; CFU: colony-forming unit; DAPI: diamidino-2-phenylindole: ECFC: endothelial colony-forming cells; EGM: endothelial growth medium; EOC: endothelial outgrowth cell; EPC: endothelial progenitor cell; G-CSF: granulocyte colony-stimulating factor; gfr: growth factor-reduced; HPC: hematopoietic progenitor cell; HUVEC: human umbilical vein endothelial cell; MNC: mononuclear cell; MSC: mesenchymal stromal (or stem) cell; NSG: NOD scid gamma; SMA: smooth muscle actin; VCAM-1: vascular cell adhesion molecule 1 (also called CD106); VEGF: vascular endothelial growth factor; vWf: von Willebrand Factor.

## Competing interests

The authors declare that they have no competing interests.

## Authors' contributions

GRB was principal investigator, designed and managed the study, devised and carried out immunohistochemistry, analysed data and wrote the manuscript. OT designed and managed the study, isolated and characterized human cells, conducted sponge implant experiments and histology (vessel density counts) and reviewed the study. KS advised and assisted with human cell isolation and sponge implant experiments. PWFH devised the sponge implant model, advised on design and reviewed the study. NLM, DEN and MLT advised on design and reviewed the study. All authors read, reviewed and approved the manuscript for publication.

## Supplementary Material

Additional file 1 **Extended set of fluorescent immunohistochemistry images of vessels stained with each antibody pair**. **Figure S1.** No primary antibodies: control. **Figure S2a.** Primary antibodies: cross-reactive rabbit anti-a-smooth-muscle actin with human-specific mouse anti-CD31. Examples of images of mouse-only vessels without incorporated human cells. **Figure S2b. ** Primary antibodies: cross-reactive rabbit anti-a-smooth-muscle actin with human-specific mouse anti-CD31. Examples of images of vessels with incorporated human cells. **Figure S3a** Primary antibodies: cross-reactive rabbit anti-CD31 with human-specific mouse anti-CD146. Examples of images of mouse-only vessels without incorporated human cells. **Figure S3b.** Primary antibodies: cross-reactive rabbit anti-CD31 with human-specific mouse anti-CD146. Examples of images of vessels with incorporated human cells. **Figure S4a** Primary antibodies: cross-reactive rabbit anti-CD105 (endoglin) with human-specific mouse anti-CD106 (VCAM-1). Examples of images of vessels showing only anti-CD105 binding. No vessels were found showing anti-CD106 binding, so vessels incorporating human cells can not be distinguished from host-only (mouse) vessels. **Figure S4b** Primary antibodies: cross-reactive rabbit anti-CD105 (endoglin) with human-specific mouse anti-CD106 (VCAM-1). Examples of images of human cells (bound by human-specific anti-CD106) which remain free in sponges and are not associated with vessels or other structures. **FigureS5a** Primary antibodies: cross-reactive rabbit anti-CD146 with human-specific mouse anti-von Willebrand factor. Examples of images of mouse-only vessels without incorporated human cells. **Figure S5b** Primary antibodies: cross-reactive rabbit anti-CD31 with human-specific. Click here for file
