# Supplementary material for: Systematic assessment in an animal model of the angiogenic potential of different human cell sources for therapeutic revascularization
Source: Stem Cell Res Ther. 2012 Jul 3;3(4):23. doi: 10.1186/scrt114 (PMC3580461; doi:10.1186/scrt114)
Supplement: Additional file 1 — Extended set of fluorescent immunohistochemistry images of vessels stained with each antibody pair. Figure S1. No primary antibodies: control. Figure S2a. Primary antibodies: cross-reactive rabbit anti-a-smooth-muscle actin with human-specific mouse anti-CD31. Examples of images of mouse-only vessels without incorporated human cells. Figure S2b. Primary antibodies: cross-reactive rabbit anti-a-smooth-muscle actin with human-specific mouse anti-CD31. Examples of images of vessels with incorporated human cells. Figure S3a Primary antibodies: cross-reactive rabbit anti-CD31 with human-specific mouse anti-CD146. Examples of images of mouse-only vessels without incorporated human cells. Figure S3b. Primary antibodies: cross-reactive rabbit anti-CD31 with human-specific mouse anti-CD146. Examples of images of vessels with incorporated human cells. Figure S4a Primary antibodies: cross-reactive rabbit anti-CD105 (endoglin) with human-specific mouse anti-CD106 (VCAM-1). Examples of images of vessels showing only anti-CD105 binding. No vessels were found showing anti-CD106 binding, so vessels incorporating human cells can not be distinguished from host-only (mouse) vessels. Figure S4b Primary antibodies: cross-reactive rabbit anti-CD105 (endoglin) with human-specific mouse anti-CD106 (VCAM-1). Examples of images of human cells (bound by human-specific anti-CD106) which remain free in sponges and are not associated with vessels or other structures. FigureS5a Primary antibodies: cross-reactive rabbit anti-CD146 with human-specific mouse anti-von Willebrand factor. Examples of images of mouse-only vessels without incorporated human cells. Figure S5b Primary antibodies: cross-reactive rabbit anti-CD31 with human-specific. [file scrt114-S1.PDF]

# Additional File

Accompanying paper:

**Systematic assessment in an animal model of the angiogenic potential of different human cell sources for therapeutic revascularisation.**

**Authors:** Barclay GR, Tura O, Samuel K, Hadoke PWF, Mills NL, Newby DE & Turner ML

## Content:

**Extended set of Immunohistochemistry Images (1 to 5b) showing examples of sponge vessel staining with all antibody pairs used, and giving examples of mouse-only (host) and human (implant) staining for each antibody pair.**

A wide range of vessels with different morphology and staining patterns with different antibodies were found during immunohistochemical examination. A range of examples are shown of captured images for each of the five antibody combinations used, and focusing on (a) mouse (host) vessels with no incorporation of human cells as demonstrated by failure to bind human-specific antibodies, and (b) vessels showing incorporation of human cells demonstrated by binding of human-specific antibodies. These are not comprehensive, but do cover the main features observed, especially with regard to clearly discriminating vessels which incorporated implanted human cells.

## Captured fluorescence microscopy images

### Figure 1.

No primary antibodies: control.

### Figure 2a.

Primary antibodies: cross-reactive rabbit anti-α-smooth-muscle actin with human-specific mouse anti-CD31. Examples of images of mouse-only vessels without incorporated human cells.

### Figure 2b.

Primary antibodies: cross-reactive rabbit anti-α-smooth-muscle actin with human-specific mouse anti-CD31. Examples of images of vessels with incorporated human cells.

### Figure 3a.

Primary antibodies: cross-reactive rabbit anti-CD31 with human-specific mouse anti-CD146. Examples of images of mouse-only vessels without incorporated human cells.

**Figure 3b.**

Primary antibodies: cross-reactive rabbit anti-CD31 with human-specific mouse anti-CD146. Examples of images of vessels with incorporated human cells.

**Figure 4a.**

Primary antibodies: cross-reactive rabbit anti-CD105 (endoglin) with human-specific mouse anti-CD106 (VCAM-1). Examples of images of vessels showing only anti-CD105 binding. No vessels were found showing anti-CD106 binding, so vessels incorporating human cells can not be distinguished from host-only (mouse) vessels..

**Figure 4b.**

Primary antibodies: cross-reactive rabbit anti-CD105 (endoglin) with human-specific mouse anti-CD106 (VCAM-1). Examples of images of human cells (bound by human-specific anti-CD106) which remain free in sponges and are not associated with vessels or other structures.

**Figure 5a.**

Primary antibodies: cross-reactive rabbit anti-CD146 with human-specific mouse anti-von Willebrand factor. Examples of images of mouse-only vessels without incorporated human cells.

**Figure 5b.**

Primary antibodies: cross-reactive rabbit anti-CD31 with human-specific mouse anti-CD146. Examples of images of vessels with incorporated human cells.

Comment is supplied in the legend below each figure.

*It is recommended that the PDF file images be viewed on a computer monitor, where PDF images are of a resolution which should allow inspection at up to 500% enlargement on any monitor without obvious pixelation. This allows inspection of the more subtle features in images, such as the relative brightness of staining of mouse-only vessel endothelial lumina and perivascular media by different cross-reactive rabbit antibodies, or punctate staining of human-vessel endothelial lumina by human-specific anti-vWf. Note that the observed staining is due to the same green-fluorochrome-conjugated goat anti-rabbit IgG secondary antibody, so differences in brightness must reflect the amounts of primary antibody present [specifically bound to various features] for the secondary antibody to bind to, reflecting different amounts of the markers expressed by different features.*

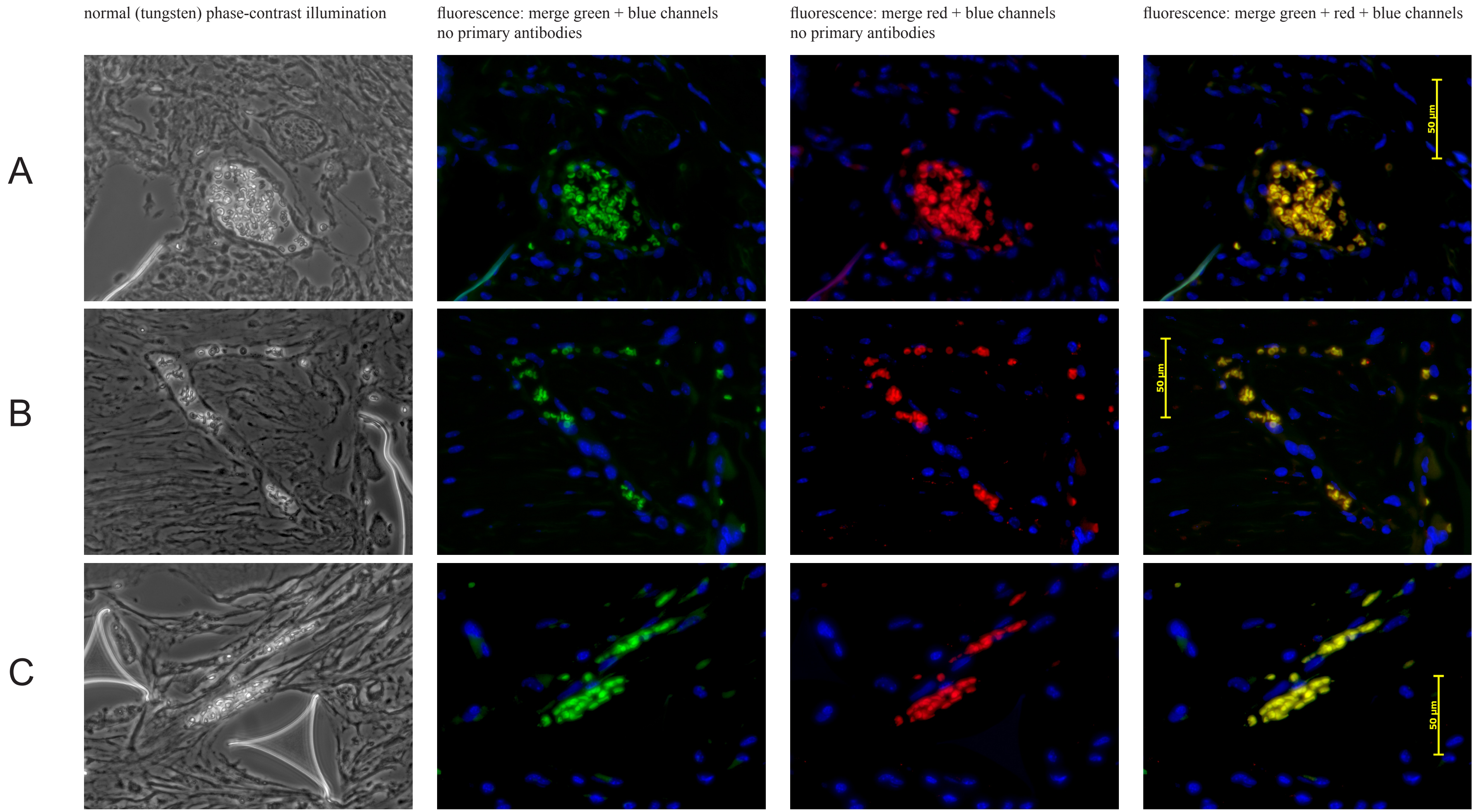


**Additional File: Figure 1.** Immunohistochemistry of vessels in sponge sections: CONTROL - no primary antibodies, secondary fluorescent goat anti-mouse IgG and goat anti-rabbit IgG, DAPI nuclear stain (blue).

There is no immunofluorescent staining of vessel walls or other cells in the absence of primary mouse or rabbit antibodies. Erythrocytes can usually be recognised in perfused vessel sections in the phase-contrast images. The only fluorescence comes from erythrocytes in perfused vessels which autofluoresce in both red and green channels, and from the blue fluorescence of DAPI stained nuclei of cells in the vessel lumen and in tissue cells between vessels, but not in erythrocytes within vessels. Erythrocytes in the merged three-colour image are yellow from the contribution of both red and green autofluoresence. Vessels images have been selected to show different sizes and shapes characteristic of the range of vessel morphology found in sponges.

Implanted cells: (A) $CD34^+$ cells enriched from cord blood MNC; (B) $CD34^+$ cells enriched from cord blood MNC; (C) pre-passage (early) HUVEC

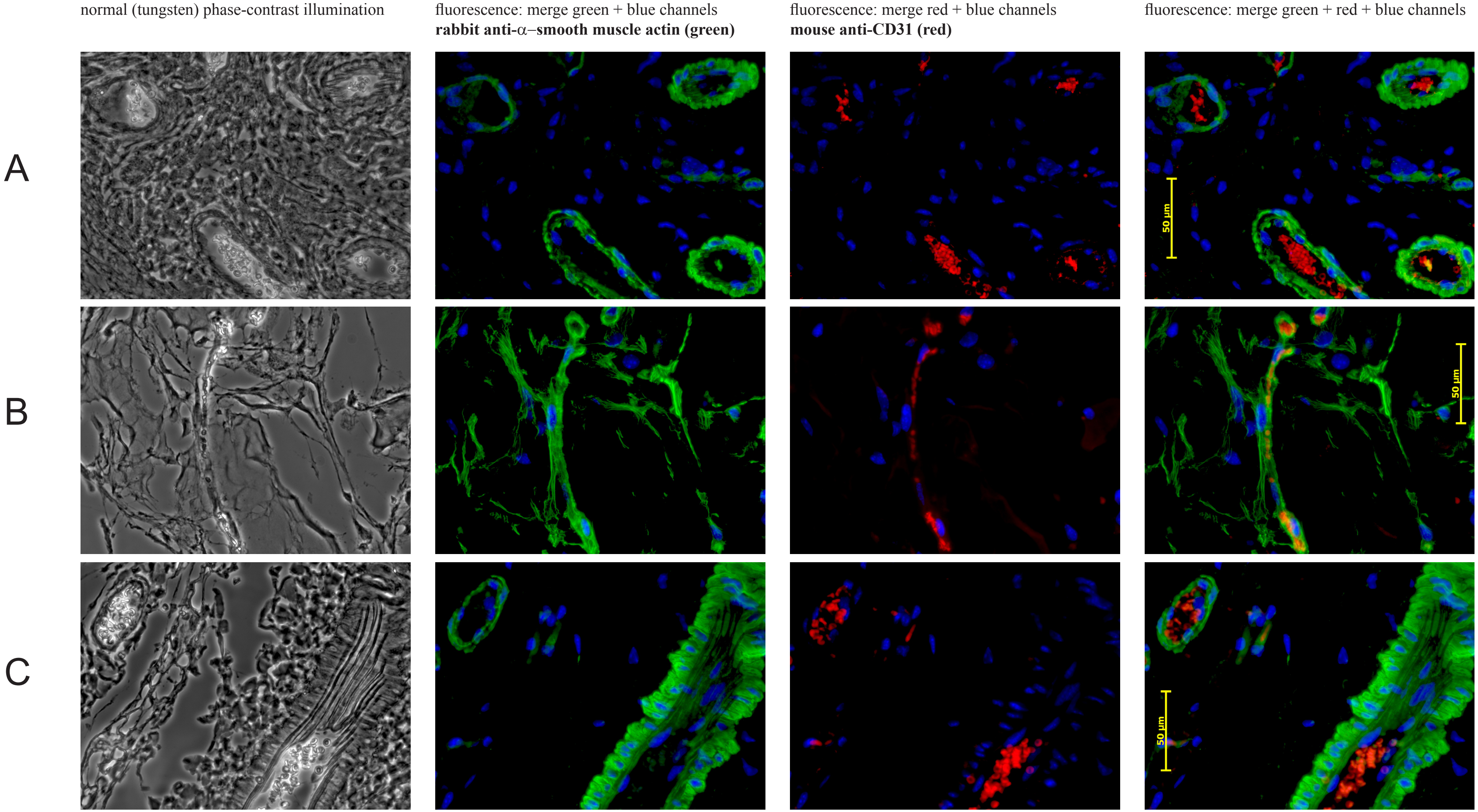


**<u>Additional File: Figure 2a.</u>** Immunohistochemistry of vessels in sponge sections. Primary antibodies: cross-reactive rabbit **anti-α-smooth muscle actin** (green) with human-specific mouse **anti-CD31** (red). Secondary fluorescent goat anti-rabbit IgG (green) with goat anti-mouse IgG (red), and DAPI nuclear stain (blue).

**Mouse vessels <u>without</u> incorporation of human cells**. Vessel walls stain with cross-reactive rabbit anti-α-smooth muscle actin but not with human-specific mouse anti-CD31. Erythrocytes in perfused vessels autofluoresce. The red autofluorescence is bright in red channel images in the absence of any red immunofluorescence, but the green autofluorescence is weak or absent in the green channel images in the presence of much brighter green immunofluorescence (compare the green immunofluorescence of rabbit anti-CD31 in Additional Fig 3a, which is weaker), so that erythrocytes are red in the merged three-colour image. Vessels show different sizes and shapes characteristic of the range of vessels found in sponges. Some vessels (rows A and C) show more than one layer of cells staining with the anti-α-SMA in the vessel walls, very evident in the large vessel in row C. We hypothesise that the outer layer may be media containing pericytes and smooth muscle cells surrounding an inner lining endothelial lumen layer. Such double-layer vessels were not uncommon, but were found only for mouse (host) vessels, not in vessels in which human cells had incorporated, and were also found to stain with all the other cross-reactive (anti-mouse) anti-endothelial antibodies tested. Some α-SMA not associated with vessels is seen in B (discussed in Additional Fig S2 legend).

Implanted cells: (A) unfractioned G-CSF-mobilised peripheral blood MNC; (B) monocyte-rich (2h plastic adherent) peripheral blood MNC; (C) monocyte-depleted (non-adherent) peripheral blood MNC.

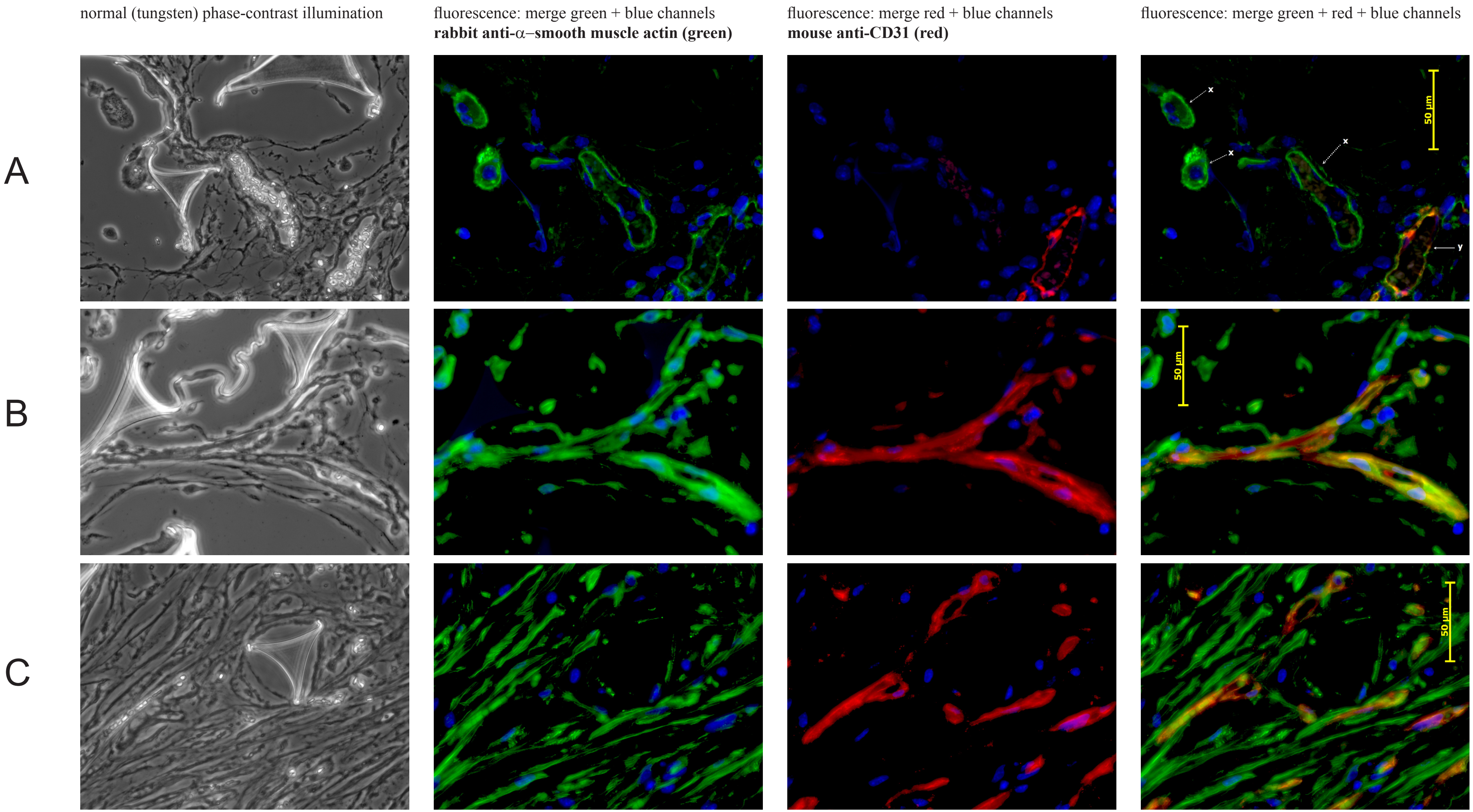


**Additional File: Figure 2b.** Immunohistochemistry of vessels in sponge sections. Primary antibodies: cross-reactive rabbit anti-α-smooth muscle actin (green) with human-specific mouse anti-CD31 (red). Secondary fluorescent goat anti-rabbit IgG (green) with goat anti-mouse IgG (red), and DAPI nuclear stain (blue).

**Vessels incorporating implanted human cells.** Vessel walls stain with cross-reactive rabbit anti-α-smooth muscle actin and with human-specific mouse anti-CD31. Erythrocytes in perfused vessels autofluoresce. This autofluorescence in both green and red channels is dim or not apparent in images in the presence of much brighter immunofluorescence in both channels. Vessels show different sizes and shapes characteristic of the range of vessels found in sponges. Some vessels shown (rows A, broken line arrows, x) stain only with cross-reactive rabbit anti-α-smooth muscle actin (green) but do not stain with the human-specific mouse anti-CD31 (red) so do not contain incorporated human cells, while other vessels in the same field (smooth line arrow, y) stain with both antibodies and are comprised of human cells. In some fields (C) there is extensive deposition of α-SMA in tissue outside vessels, which was commonly found in discrete areas of most sponge sections, so binding of this cross-reactive anti-α-smooth muscle actin antibody is not strictly vessel specific. However other factors (morphology, erythrocyte perfusion) contribute to the interpretation as to whether it labels a vessel or other tissue. It also appears that the anti-α-smooth muscle actin antibody labels endothelial lumen as well as cells in the surrounding media (see Additional Fig 2a). None of these vessels with human cells showed substantial media.

Implanted cells: (A) EOC from cord blood; (B) EOC from cord blood; (C) pre-passage (early) HUVECs.

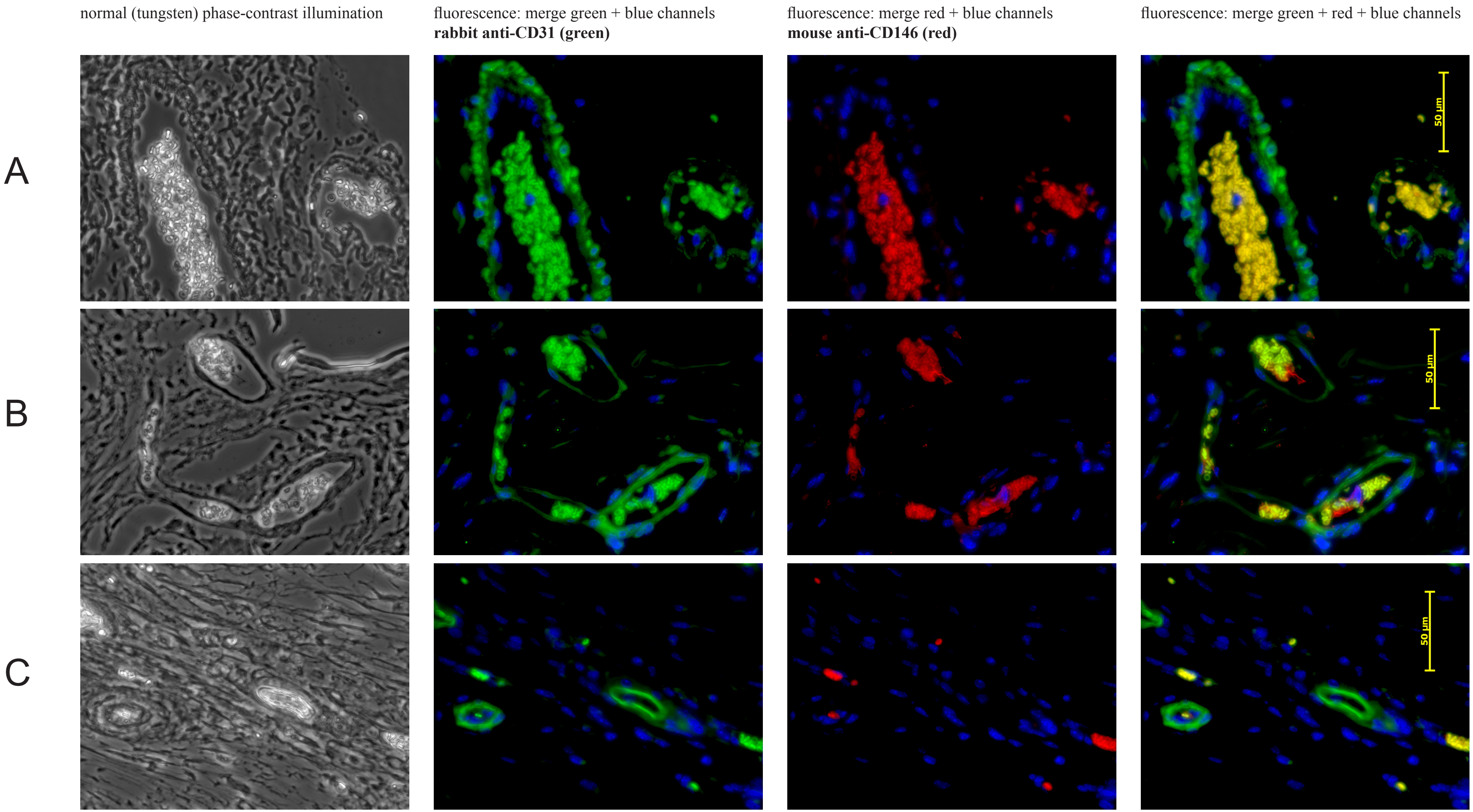


**Additional File: Figure 3a.** Immunohistochemistry of vessels in sponge sections. Primary antibodies: cross-reactive **rabbit anti-CD31 (green)** with human-specific **mouse anti-CD146 (red)**. Secondary fluorescent goat anti-rabbit IgG (green) with goat anti-mouse IgG (red), and DAPI nuclear stain (blue).

**Mouse vessels without incorporation of human cells.** Vessel walls stain with cross-reactive rabbit anti-CD31 but not with human-specific mouse anti-CD146. Erythrocytes in perfused vessels autofluoresce. Red erythrocyte autofluorescence is bright in red channel images in the absence of red immunofluorescence; the green autofluorescence is as bright or brighter than the anti-CD31 green immunofluorescence on mouse vessels in the green channel images (contrast Fig 2a where there is little erythrocyte green autofluorescence). In general this anti-CD31 antibody labels mouse vessels more weakly than the other antibodies used, which must reflect less bound antibody since the same fluorescent goat anti-rabbit IgG is used with all the cross-reactive rabbit antibodies to demonstrate binding. Vessels show different sizes and shapes characteristic of the range of vessels found in sponges. The large vessel on the left in the first row (A) appears to show more than one layer of cells staining with the anti-CD31 in the vessel walls, and examples were also found for mouse-only vessels with all the other antibody combinations. Here the outer cell layer (putative media pericytes) appears to stain brighter than the inner cell layer (putative luminal endothelial cells).

Implanted cells: (A) $CD34^+$-depleted cord blood MNC; (B) $CD34^+$-enriched cord blood MNC; (C) unfractioned bone marrow MNC

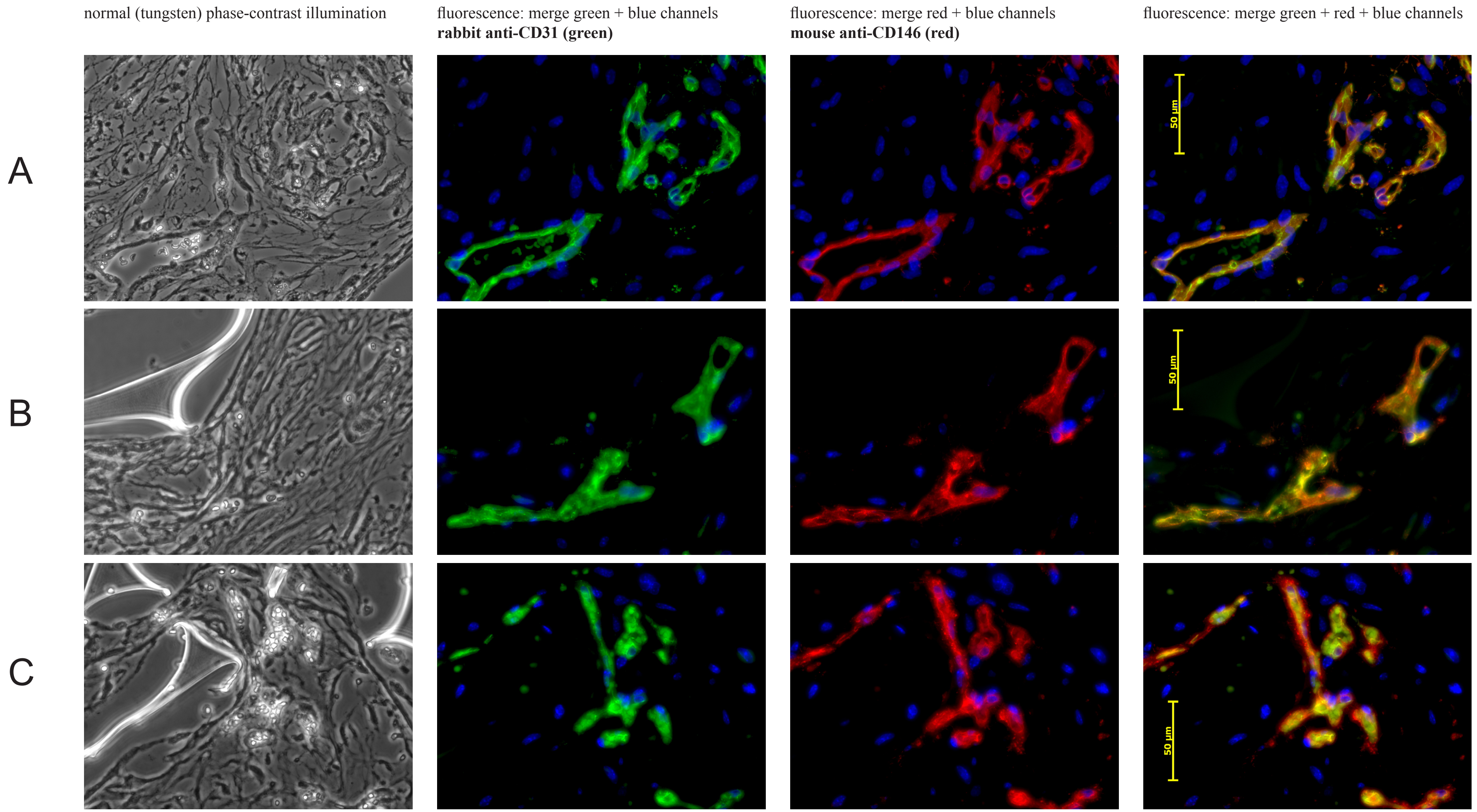


**<u>Additional File: Figure 3b.</u>** Immunohistochemistry of vessels in sponge sections. Primary antibodies: cross-reactive **rabbit anti-CD31 (green)** with human-specific mouse **anti-CD146 (red)**. Secondary fluorescent goat anti-rabbit IgG (green) with goat anti-mouse IgG (red), and DAPI nuclear stain (blue).

**Vessels <u>incorporating</u> implanted human cells**. Vessel walls stain with cross-reactive rabbit anti-CD31 (green) and with human-specific mouse anti-CD146 (red). Erythrocytes in perfused vessels autofluoresce. Relative to immunofluorescence brightness the autofluorescence in the green channel is apparent but the autofluorescence in the red channels is dim or not apparent in images. This effect on captured images is characteristic of all the human-specific red immunofluorescent antibody binding and the dimming of erythrocyte red autofluorescence in images in its presence helps confirm specific anti-human antibody binding when human cells are incorporated into vessels. The retention of some green erythrocyte autofluoresence in images may reflect the relatively weak binding of the cross-reactive rabbit anti-CD31, also seen on mouse-only vessels (Fig 3a) where the relative erythrocyte green autofluoresence is even brighter (possibly indicating weaker binding of this anti-CD31 to mouse vessel cells than to human vessel cells). Vessels show different sizes and shapes characteristic of the range of vessels found in sponges. None of the vessels with human cells show an evident medial layer (compare Fig 3a, A), and appear to express only endothelial lumen.

Implanted cells: (A) EOC from cord blood MNC; (B) pre-passage (early) HUVECs; (C) EOC from foetal liver MNC.

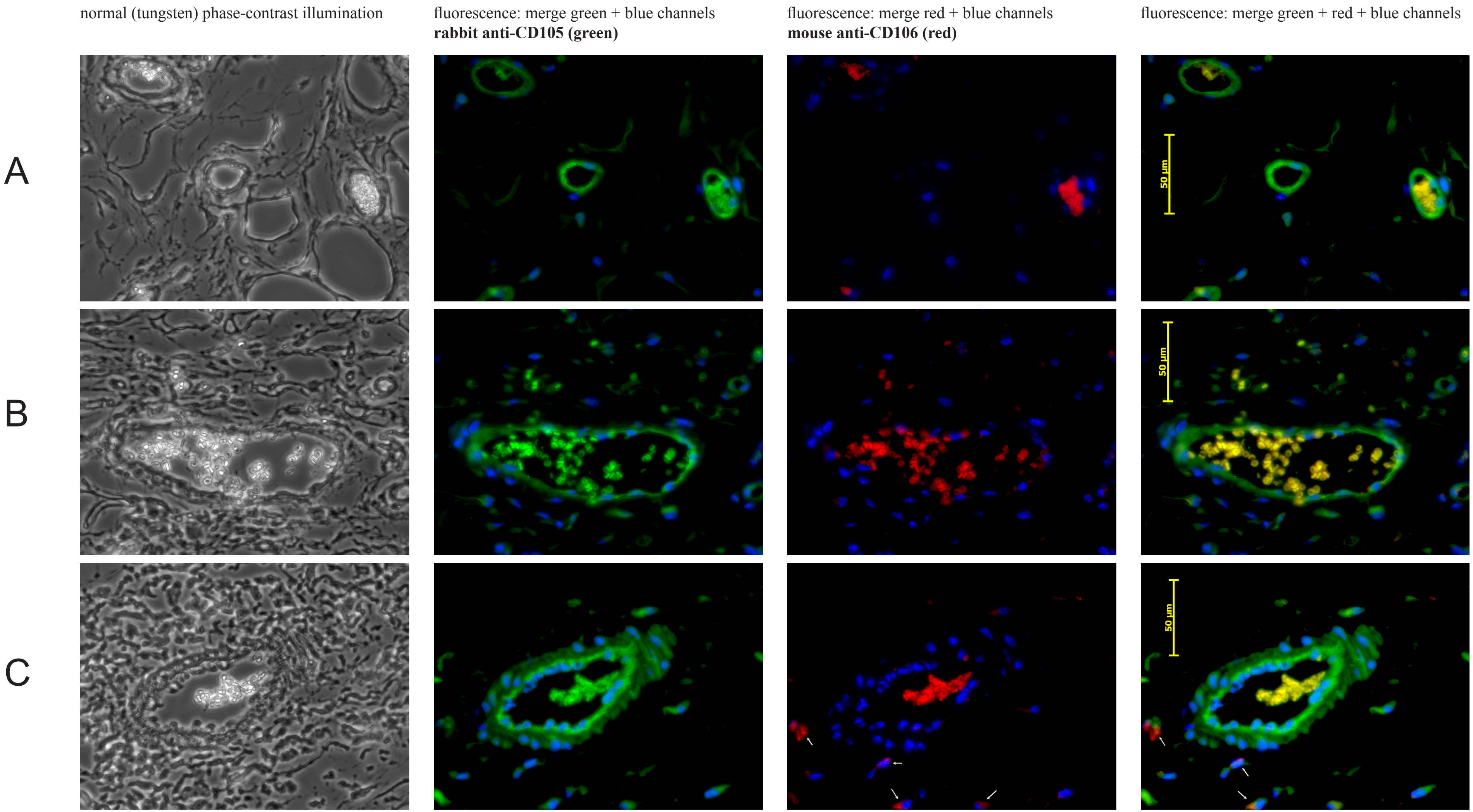


**<u>Additional File: Figure 4a.</u>** Immunohistochemistry of vessels in sponge sections. Primary antibodies: cross-reactive rabbit **anti-CD105 (endoglin)** (green) with human-specific **mouse anti-CD106** (VCAM-1) (red). Secondary fluorescent goat anti-rabbit IgG (green) with goat anti-mouse IgG (red), and DAPI nuclear stain (blue).

***Probable* mouse vessels <u>without</u> incorporation of human cells.** Vessel walls stain with cross-reactive rabbit anti-CD105 but not with human-specific mouse anti-CD106. Erythrocytes in perfused vessels autofluoresce. This autofluorescence is as bright or brighter than immunofluorescence in both green and red channels in images. In general the anti-CD105 antibody labels mouse vessels diffusely but weakly. No vessels were detected which bound anti-CD106 in any sponge, including those sponges in which human cells in vessels were detected by other antibodies (e.g. when EOC were implanted), so it is concluded that this mouse anti-CD106 does not bind to vessels. However this anti-CD106 binds to other human cells not incorporated in vessels (see Fig 4b): some examples may be seen in C (arrowed). Since no vessels were detected by other human-specific antibodies with the cells in the sponges shown above, these images probably represent mouse-only vessels. However species origin of vessels cannot be directly identify with this anti-CD105 + anti-CD106 antibody combination. The anti-CD105 (endoglin) antibody did detect some double-layer vessel walls (C) where the inner cells (putative luminal endothelial) were more brighly labelled than the outer cells (putative media pericytes), in contrast to the other antibody combinations used (for example, see Fig 3a,A where putative pericytes label brighter than endothelial cells with anti-CD31).

Implanted cells: (A) unfractioned bone marrow MNC; (B) $CD34^{+}$-enriched bone marrow MNC; (C) $CD34^{+}$-depleted cord blood MNC.

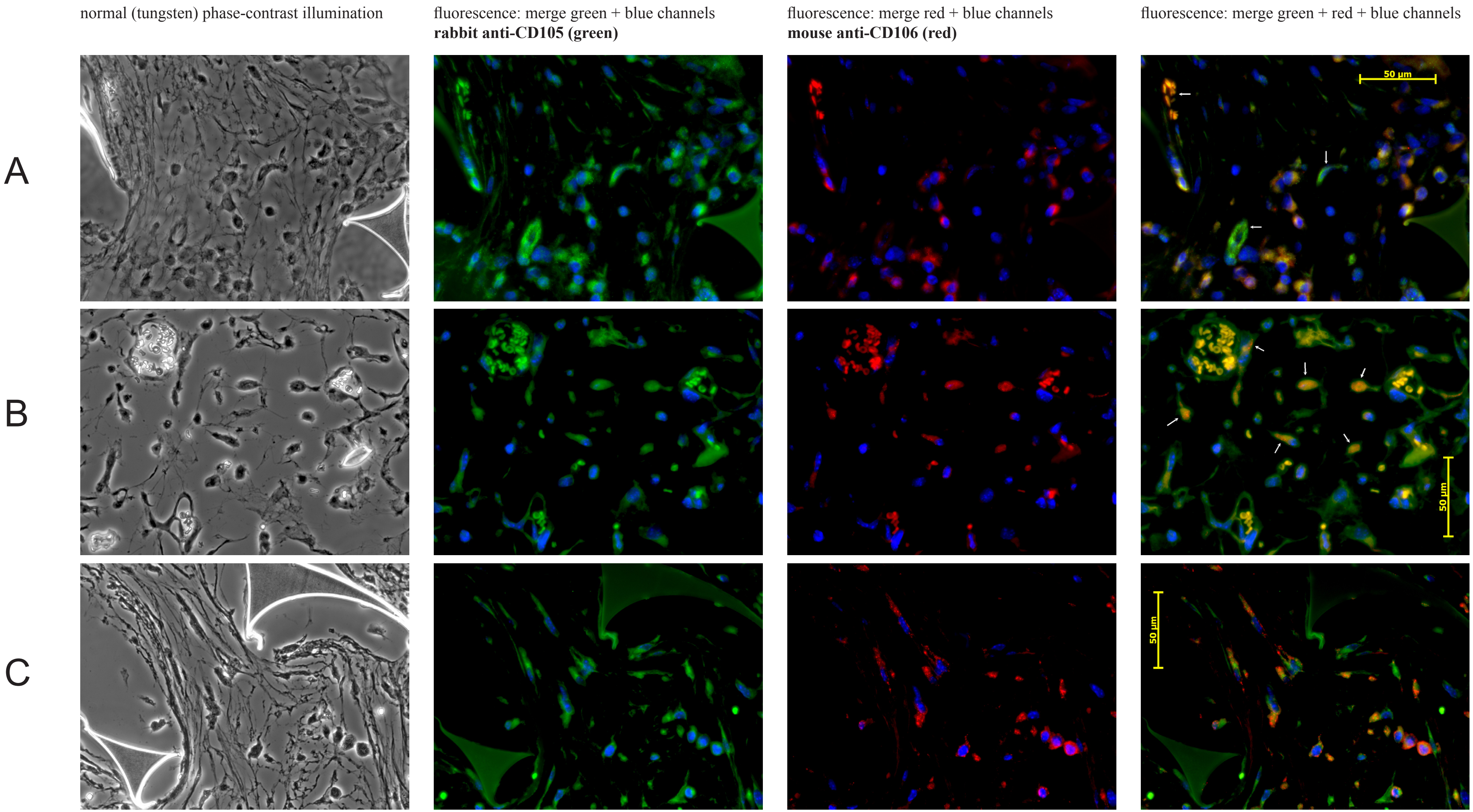


**Additional File: Figure 4b.** Immunohistochemistry of vessels in sponge sections. Primary antibodies: cross-reactive **rabbit anti-CD105 (endoglin)** (green) with human-specific **mouse anti-CD106 (VCAM-1)** (red). Secondary fluorescent goat anti-rabbit IgG (green) with goat anti-mouse IgG (red), and DAPI nuclear stain (blue).

**Free human cells (not in vessels).** Single cells, not in structures, were stained with cross-reactive rabbit anti-CD105 and with human-specific mouse anti-CD106. Erythrocytes in perfused vessels autofluoresce (A and B). Vessels are marked in A (arrows) and examples of dual-staining single human cells are marked in B (arrows). Such single cells are found when monocytes are implanted (e.g. monocyte-enriched or unfractioned MNC) but not when monocytes are absent (e.g. EOC, $CD34^{+}$-enriched MNC, HUVECs). They are human since they stain with human-specific anti-CD106 and were also found with human-specific anti-CD146 and anti-vWf, co-staining with cross-reactive CD31 and CD105 (e.g. see Figure 2d, main text), and are interpreted as monocytes which have differentiated following implant to up-regulate expression of these endothelial-associated markers. They do not appear to be associated with vessels or other tissue structures. This human-specific mouse anti-CD106 (VCAM-1) failed to stain any human vessels in sponges where vessels were identified by other human-specific antibodies (e.g. where EOC were implanted).

Implanted cells: (A) monocytes (plastic-adherent peripheral blood MNC, > 80% $CD14^{+}$ monocytes); (B) monocytes (plastic-adherent peripheral blood MNC, > 80% $CD14^{+}$ monocytes); (C) unfractioned G-CSF-mobilised peripheral blood MNC (~ 20% $CD14^{+}$ monocytes).

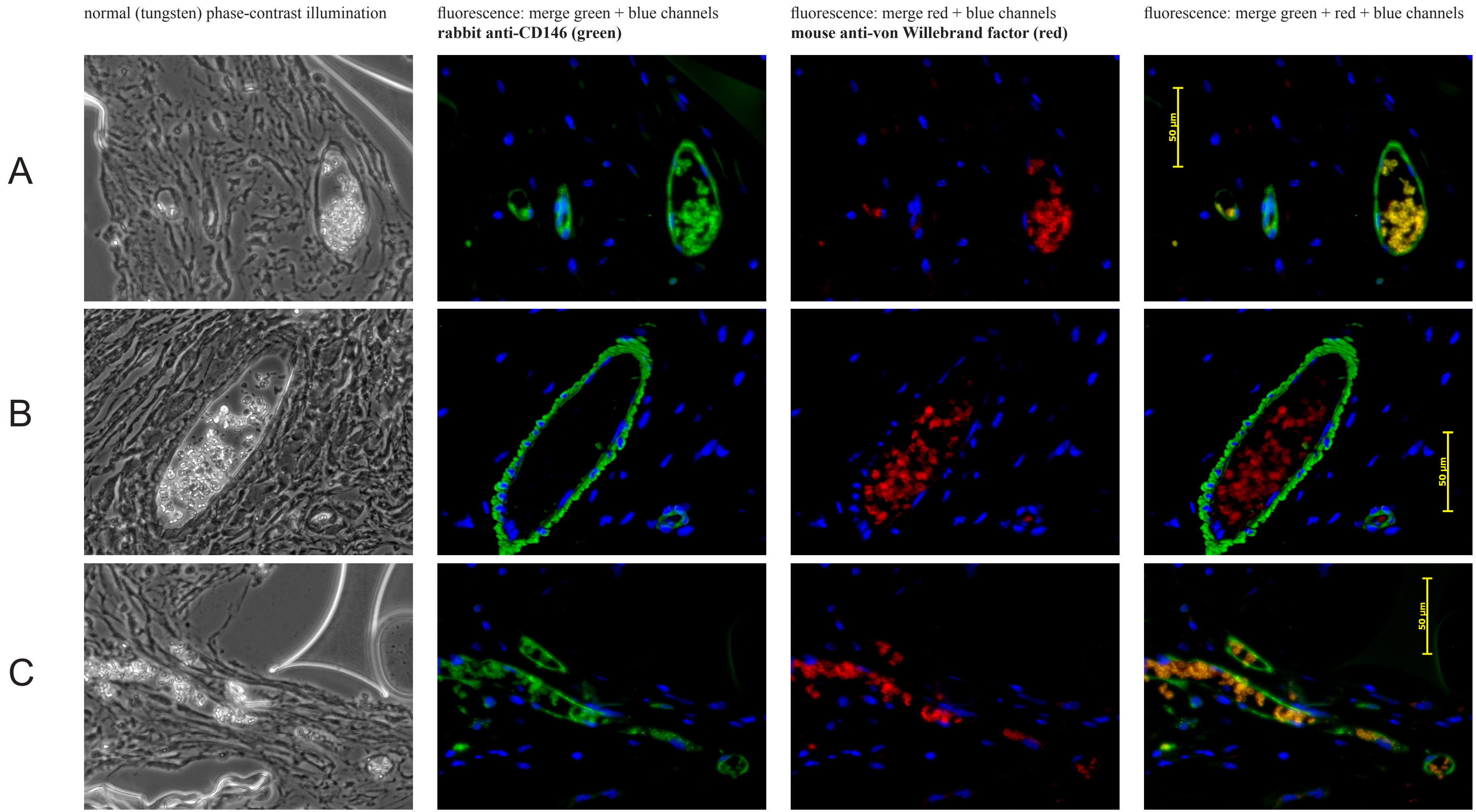


**Additional File: Figure 5a.** Immunohistochemistry of vessels in sponge sections. Primary antibodies: cross-reactive **rabbit anti-CD106** (green) with human-specific **mouse anti-von Willebrand factor (vWf)** (red). Secondary fluorescent goat anti-rabbit IgG (green) with goat anti-mouse IgG (red), and DAPI nuclear stain (blue).

**Mouse vessels without incorporation of human cells.** Vessel walls stain with cross-reactive rabbit anti-CD146 but not with human-specific mouse anti-vWf. Erythrocytes in perfused vessels autofluoresce. The autofluorescence is visible in the red channel in all images in the absence of any red immunofluorescence, and in the green channel is as bright as immunofluorescence in images A and C but not in B. In B the anti-CD146 detects a double-layer vessel wall where the outer cells (putative media pericytes) are more brightly labelled then the luminal endothelial cells lining the vessel, and diminish the relative erythrocyte green autofluorescence in the captured image (compared to A and C) so that erythrocytes appear red in the merged three-colour image in B solely from red autofluorescence where there is no contribution from green autofluorescence, but appear yellow/orange in A and C where there is a contribution to their merged colour from green autofluorescence.

Implanted cells: (A) $CD34^+$-enriched bone marrow MNC; (B) unfractioned mobilised peripheral blood MNC; (C) $CD34^+$-enriched bone marrow MNC.

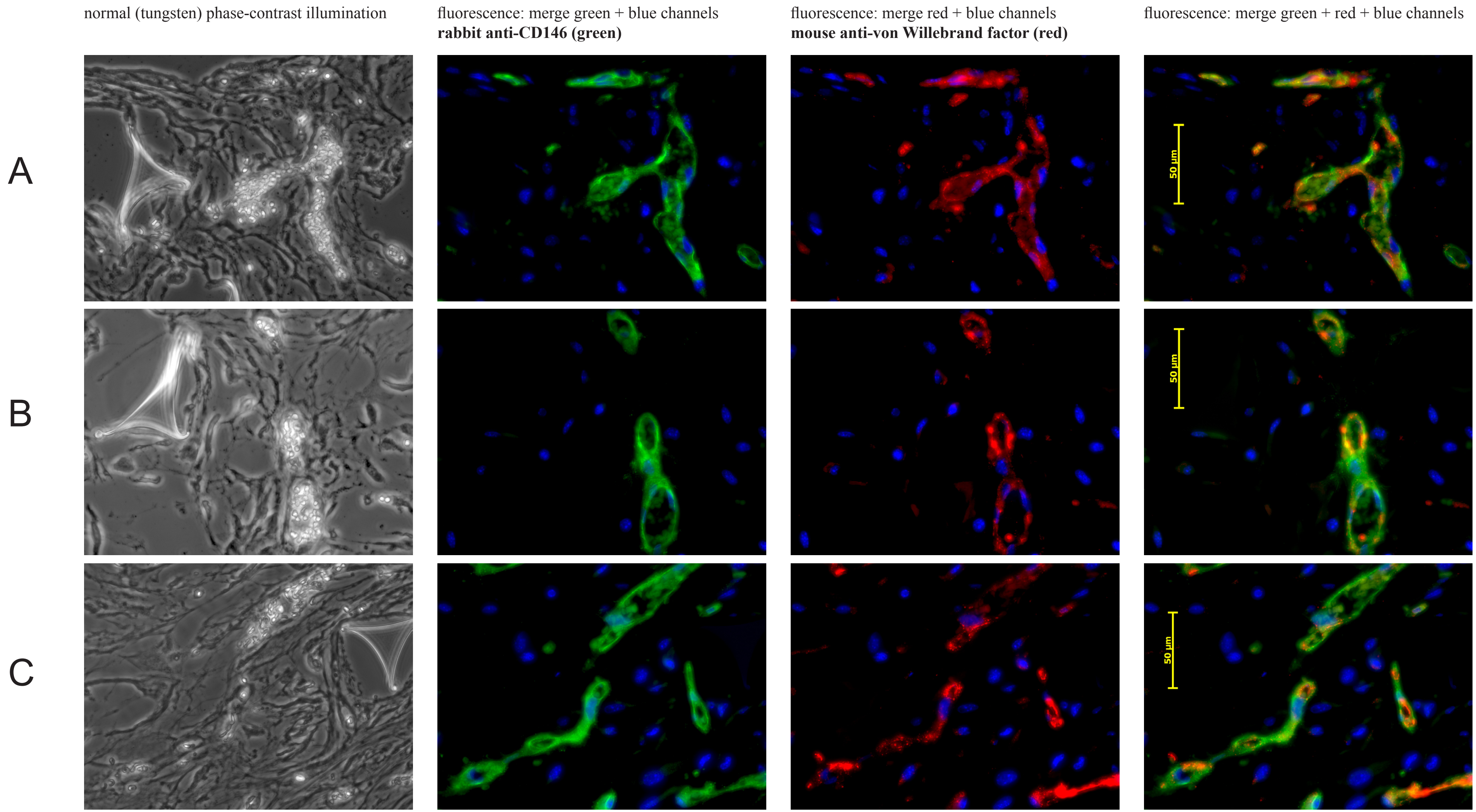


**<u>Additional File: Figure 5b.</u>** Immunohistochemistry of vessels in sponge sections. Primary antibodies: cross-reactive **rabbit anti-CD106** (green) with human-specific **mouse anti-von Willebrand factor (vWf)** (red). Secondary fluorescent goat anti-rabbit IgG (green) with goat anti-mouse IgG (red), and DAPI nuclear stain (blue).

**Vessels <u>incorporating</u> implanted human cells**. Vessel walls stain with cross-reactive rabbit anti-CD146 and with human-specific mouse anti-vWf. Erythrocytes in perfused vessels autofluoresce. Relative to immunofluorescence, the autofluorescence in the green channel is weak but apparent in images, but the autofluorescence in the red channel is very dim or not apparent in images due to the bright anti-vWf immunofluorescence. The red immunofluorescence of the anti-vWf is punctate, appearing as bright spots on a weaker more diffusely-stained background. The anti-vWf binding is more evident alone, in the two-colour image merged with DAPI-stained nuclei (column 3), rather than in the three-colour image (column 4) where the more homogeneously distributed binding of anti-CD146 (green) masks the weak duffuse anti-vWf binding and only the bright punctate anti-vWf patches appear, as yellow/orange spots of anti-vWf on the green background of anti-CD146 in the merged image.

Implanted cells: (A) EOC; (B) EOC; (C) pre-passage (early) HUVECs.
